# Characterization of wooden breast myopathy: a focus on syndecans and ECM remodeling

**DOI:** 10.3389/fphys.2023.1301804

**Published:** 2023-12-05

**Authors:** Lucie Pejšková, Sissel Beate Rønning, Matthew Peter Kent, Nina Therese Solberg, Vibeke Høst, To Thu-Hien, Jens Petter Wold, Marianne Lunde, Ellen Mosleth, Addolorata Pisconti, Svein Olav Kolset, Cathrine Rein Carlson, Mona Elisabeth Pedersen

**Affiliations:** ^1^ Raw Materials and Optimization, Nofima AS, Ås, Norway; ^2^ Center for Integrative Genetics, Department of Animal and Aquacultural Sciences, Faculty of Biosciences (BIOVIT), Norwegian University of Life Sciences (NMBU), Ås, Norway; ^3^ Institute for Experimental Medical Research, Oslo University Hospital and University of Oslo, Oslo, Norway; ^4^ Department of Biochemistry and Cell Biology, Stony Brook, NY, United States; ^5^ Department of Nutrition, Institute of Basic Medical Science, University of Oslo, Oslo, Norway

**Keywords:** wooden breast, myopathy, syndecans, extracellular matrix, broiler chicken

## Abstract

**Introduction:** The skeletal muscle deformity of commercial chickens *(Gallus gallus)*, known as the wooden breast (WB), is associated with fibrotic myopathy of unknown etiology. For future breeding strategies and genetic improvements, it is essential to identify the molecular mechanisms underlying the phenotype. The pathophysiological hallmarks of WB include severe skeletal muscle fibrosis, inflammation, myofiber necrosis, and multifocal degeneration of muscle tissue. The transmembrane proteoglycans syndecans have a wide spectrum of biological functions and are master regulators of tissue homeostasis. They are upregulated and shed (cleaved) as a regulatory mechanism during tissue repair and regeneration. During the last decades, it has become clear that the syndecan family also has critical functions in skeletal muscle growth, however, their potential involvement in WB pathogenesis is unknown.

**Methods:** In this study, we have categorized four groups of WB myopathy in broiler chickens and performed a comprehensive characterization of the molecular and histological profiles of two of them, with a special focus on the role of the syndecans and remodeling of the extracellular matrix (ECM).

**Results and discussion:** Our findings reveal differential expression and shedding of the four syndecan family members and increased matrix metalloproteinase activity. Additionally, we identified alterations in key signaling pathways such as MAPK, AKT, and Wnt. Our work provides novel insights into a deeper understanding of WB pathogenesis and suggests potential therapeutic targets for this condition.

## 1 Introduction

The chicken skeletal myopathy, commonly known as the wooden breast (WB), is a condition that affects the breast muscles (*Pectoralis major*) of fast-growing broilers selected for meat production purposes. It is characterized by the hardening and stiffness of the breast muscles, resulting in poor meat quality and sensory traits (Mudalal et al., 2015; Petracci and Berri, 2017; Xing et al., 2020; Oliveira et al., 2021). WB has been reported in the *Pectoralis major* muscle in predominantly fast-growing broiler lines (Sihvo et al., 2014) and is thought to be caused by the intensive genetic selection and feeding optimization that have resulted in faster growth rates and increased breast meat yields recorded worldwide over the past few decades (Petracci et al., 2015). However, the etiology of WB myopathy is unknown, and investigating the mechanisms underlying this muscle disorder is challenging. Different production regimes and feeding strategies have been tested to ameliorate the outcome of WB, but with limited success (Kuttappan et al., 2021). An especially intriguing, unanswered question is why some animals from the same breed, feeding regime, and production conditions develop WB, whereas others do not. Identifying molecular mechanisms and signaling systems involved in WB pathogenesis is important to designing future breeding strategies and could even identify therapeutic targets in modern agricultural production.

The pathophysiological hallmarks of WB include severe skeletal muscle fibrosis, inflammation, myofiber necrosis, and multifocal degeneration of muscle tissue. Fibrosis, in general, is characterized by extensive extracellular matrix (ECM) remodeling and increased depositions of ECM, especially of collagens, fibronectin 1, and small leucine-rich proteoglycans (SLRPs) (Sihvo et al., 2014). In WB myopathy, the SLRP decorin is involved in collagen organization and crosslinking (Velleman et al., 2017; Velleman, 2020). The main trigger of fibrosis, transforming growth factor-beta 2 (TGF-β2) and its SMAD signaling pathway, is upregulated in WB (Xing et al., 2021b); however, the potential involvement of other signaling pathways is still unknown.

Syndecans are key regulators of the TGF-β/Smad signaling pathway (Cui et al., 2020). The syndecan family includes four members (syndecan-1, -2, -3, and -4) that are differentially expressed in various tissues and cell types (Lambaerts et al., 2009; Velleman, 2012). Syndecans play critical roles in cell adhesion, proliferation, differentiation, migration, and survival (Couchman et al., 2015) and are important for skeletal muscle growth, health, and aging (Pisconti et al., 2012; Rønning et al., 2015; Pisconti et al., 2016; Velleman and Song, 2017; Rønning et al., 2020; Sztretye et al., 2023). They also play a role in ECM remodeling and can act as co-receptors for growth factors, chemokines, and extracellular matrix molecules (Xian et al., 2009; Couchman, 2010; Lunde et al., 2016). Syndecans are composed of an extracellular domain (ectodomain), a well-characterized transmembrane region, and a highly conserved short cytoplasmic domain. The ectodomains of all syndecans harbor heparan sulfate (HS) chains, and in the case of syndecan-1 and -3, they also harbor chondroitin sulfate (CS) chains. Syndecans are remarkable due to their ability to regulate multiple biological activities at multiple levels: i) extracellularly, through both the glycosaminoglycan (GAG) chains and the core protein that interacts with matrix components and growth factors; ii) at the transmembrane level, via dimerization and intramembrane cleavage; iii) at the intracellular level, via interaction with cytoskeleton proteins and various intracellular signaling transducers (Couchman et al., 2015); and iv) systemically, through shedding and release of the extracellular domain by matrix metalloproteinases (MMPs) and A disintegrin and metalloproteinase with thrombospondin (ADAMTS) motifs, which can act both at the autocrine and paracrine levels as well as systemically through the bloodstream. Syndecan shedding is a physiological process; however, under different pathological conditions, shedding is increased (Manon-Jensen et al., 2010). The soluble ectodomains of syndecans can function both as promoters of signaling and as competitive inhibitors, depending on the circumstances (Manon-Jensen et al., 2010), often depending on the activity of the protein partners attached to the HS chains (Pap and Bertrand, 2013). Dysregulation of syndecan expression and shedding has been linked to various diseases, including cancer, fibrosis, and neurodegeneration (Choi et al., 2011; Gopal, 2020). MMPs and sheddases, in general, are activated by the cleavage of an inactive pro-form and can degrade ECM proteins through the cleavage of specific peptide bonds (Hrabia et al., 2019). Their activities are further regulated by tissue inhibitors of metalloproteinases (TIMPs), which can bind to the active site of MMPs, preventing their catalytic activity and the degradation of ECM proteins and shedding processes.

Few studies have addressed ECM remodeling or the involvement of syndecan family in WB myopathy, and thus, syndecans’ potential involvement in skeletal muscle fibrosis in WB is at present unknown. Importantly, transcription analysis of WB myopathy has shown upregulation of MMPs, but their protein or activity levels have not been investigated (Xing et al., 2021b). In this study, we report a thorough characterization of WB-associated fibrosis and ECM remodeling, with a particular focus on syndecans and their shedding during WB myopathy.

## 2 Materials and methods

### 2.1 Animal sampling and sample preparation

Male Ross 308 broiler chickens (*Gallus gallus*) were fed an *ad libitum* diet of pelleted wheat/maize from 10 days of age. The broiler chickens were housed in 2.4 × 0.95 m pens on wood shavings in a room with 6 h of light and 18 h of darkness and a temperature gradually reduced from 28°C to 21°C. To identify individuals with or without WB, a veterinary inspection of the hardness of breast muscle of 60 36-day post-hatching male Ross 308 broiler chickens was performed (from a larger flock *n* = 2,000, approximately 10% developed WB). Palpation of the breast muscle (*Pectoralis major*) after slaughtering was included in this pre-evaluation to confirm the grouping of affected and non-affected chickens. Further grouping of samples was performed by histology and near-infrared (NIR) spectroscopy, as described in the following section. Samples for quantitative polymerase chain reaction (qPCR), RNA sequencing (RNA-seq), Western blotting, and zymography were excised immediately after slaughter, snap-frozen in liquid nitrogen, and stored at −80°C until further analysis. For the microscopy analysis, tissue samples measuring approximately 8 mm × 8 mm × 2 mm were cut from the outer layer in the upper part of *Pectoralis major* of all animals after slaughtering and fixed in IHC Zinc Fixative (#550523, BD Pharmingen, New Jersey, United States) for 24 h before dehydrating and paraffin embedding. All animals were first sorted by palpation and then classified by histology and NIR spectroscopy. The muscle tissues used are extracted from already slaughtered chickens (Ross 308 breed, NMBU, Norway). These chickens are in line with common regulatory roles of food production, and therefore do not require REC or NSD approval. In compliance with Norwegian law regulations concerning the experimental use of animals (FOR-2015-06-18-761 §2a), ethical approval is not necessary when samples are collected from slaughtered animals/non-experimental agriculture and aquaculture. This is also confirmed by direct communication with the Norwegian Food Safety Authority (Mattilsynet).

### 2.2 Histology and immunohistochemistry

To perform histological evaluation, 3-μm-thick sections of fixed, paraffin-embedded samples were cut on a microtome (Leica RM 2165, Leica Biosystems Nussloch GmbH, Germany), mounted on poly-L-lysine-coated glass-slides, and all 60 samples were stained with hematoxylin and eosin (H&E) according to standard procedures. All slides were scanned using Aperio CS2 (Leica Biosystems Nussloch GmbH, Buffalo Grove, IL, United States), and digital images were taken using ImageScope 64 (Leica Biosystems Nussloch GmbH, Buffalo Grove, IL, United States). Picrosirius red staining was performed according to the protocol (#25901 Polysciences, Inc., PA, United States). Slides were examined using a Leica light microscope (DM6000 B, Leica Biosystems Nussloch GmbH, Germany), and images were captured with or without a polarization filter using the Leica Application Suite and DMC 4500 (Leica Microsystems, Leica Biosystems Nussloch GmbH, Germany). The H&E staining was used to classify the samples according to the severity of fibrosis into mild (*n* = 13), moderate (*n* = 22), severe (*n* = 12), and fatty (*n* = 13).

To perform immunohistochemistry analyses, tissue sections of severe and mild WB samples (*n* = 3 for each group) were fixed with 4% PFA for 10 min and washed three times in PBS–Tween (PBS–T), followed by permeabilization with 0.1% Triton X-100 for 10 min. Samples were blocked in 1x blocking buffer (#ab126587, Abcam, United Kingdom) in PBS–T for 30 min and then stained using NucBlue Live Cell Stain Ready Probe (Hoechst 33342, Invitrogen, MA, United States) and wheat germ agglutinin (WGA) (Alexa Fluor™ 488 conjugate) (#A32723, 1:200, Thermo Fisher Scientific, MA, United States) for 2 h at room temperature (RT). The samples were washed once with PBS before being transferred onto a microscope slide and mounted using the fluorescent mounting medium (#S3023, DAKO, Denmark). The sections were examined by fluorescence microscopy analysis (ZEISS Axio Observer Z1 microscope, Jena, Germany), and images were processed using ImageJ software (NIH, MD, United States).

### 2.3 Near-infrared spectroscopy

NIR spectroscopy is a rapid and non-destructive technique that can be used to grade the severity of the WB syndrome in chicken breasts. The method provides a detailed measure of the amount of protein and the degree of water binding in the breast tissue, well known to be markers of WB (Wold et al., 2017; Wold et al., 2019). The degree of water binding is constituted by a shift in the absorption peak of water at approximately 980 nm. This is the dominant variation component in spectra from chickens with large variation in the degree of WB and can easily be quantified by principal component analysis (PCA). A prototype spectroscopic instrument (Wold et al., 2020) was used to collect near-infrared spectra measured in the interactance mode. Interactance ensures measurement in the depth of the chicken breasts, typically 10–12 mm deep, which is important for proper grading of WB. A halogen light source of 50 W was used to illuminate the sample in two rectangular regions of approximately 2 mm × 20 mm. The distance between the two illuminated regions was 24 mm. Between the two illumination regions, there was a 4 × 4 mm field of view that was measured using a spectrometer, as described in detail by Wold et al. (2020). The spectra consisted of 20 evenly spaced wavelengths in the region of 761–1081 nm. For all samples (*n* = 60), one spectrum was collected in the rostral breast region after cooling to 4°C. Each measurement took 1 s and was done without contact with the sample.

### 2.4 RNA sequencing and analysis

Total RNA was extracted from muscle samples from all the individuals (*n* = 60) and stored at −80°C using the RNAdvance Tissue Kit (Beckman Coulter, IN, United States) according to the manufacturer’s instructions. RNA purity and concentration were assessed for all samples using a NanoDrop spectrophotometer (Thermo Fisher Scientific, MA, United States), and the integrity of nine random samples was assessed using a 4150 TapeStation with RNA ScreenTape (Agilent, CA, United States). Extracted RNA (1.5–4.5 μg) was sent to a commercial sequencing provider (Novogene, United Kingdom), where quality was re-assessed (lowest RIN = 9.1). Sequencing libraries were prepared using NEBNext Ultra Directional RNA Library Prep Kits (NEB, MA, United States) and sequenced (PE150) on NovoSeq 6000 instruments using S4 flow cells (Illumina, CA, United States). FastQC (Andrews, 2010) was used to assess read quality, and raw reads were trimmed using fastp (Chen et al., 2018) to remove the adapter sequence. Gene expression levels were calculated by quantifying transcript expression with Salmon (Patro et al., 2017) (using the GRCg7b reference genome from NCBI) and then summarized into gene-level data. After obtaining read counts for each gene on each sample, DESeq2 (Love et al., 2014) was used to analyze differentially expressed genes between affected and normal groups. Gene Ontology (GO) and Kyoto Encyclopedia of Genes and Genomes (KEGG) pathway enrichment analyses were estimated using the R package clusterProfiler (Yu et al., 2012; Wu et al., 2021) for upregulated and downregulated gene sets separately.

### 2.5 RNA extraction and real-time quantitative PCR

Total RNA was extracted from eight chickens classified with severe WB and eight classified with mild WB using the RNeasy Midi Kit (#75144, Qiagen, Germany) according to the manufacturer’s instructions. Approximately 100 mg of tissue was homogenized in RLT buffer containing 0.04 M DTT using the Precellys Lysing Kit (#P000911-LYSKO-A.0, Bertin Technologies, France), 4 × 20 s at 6,000 rpm with a 10-s break between shakes, followed by a 10-min spin at 5,000 g. Samples were incubated with proteinase K (#19131, Qiagen, Germany) according to the manufacturer’s instructions. cDNA was generated from 2 µg of total RNA using TaqMan reverse transcription reagents (#N8080234, Thermo Fisher Scientific, MA, United States) in a 40 µL reaction volume with random hexamers according to the manufacturer’s protocol. Real-time quantitative polymerase chain reaction (RT-qPCR) analysis was carried out using the TaqMan Gene Expression Master Mix (#4369510, Life Technologies, Thermo Fisher Scientific) and the QuantStudio 5 PCR System (Applied Biosystems, Foster City, CA, United States). The amplification protocol was initiated at 50°C for 2 min, followed by denaturation at 95°C for 10 min, 40 cycles of denaturation at 95°C for 15 min, and annealing of TaqMan probes and amplification at 60°C for 1 min. RT-qPCR analyses were performed with three technical replicates from each sample. The relative gene expression (RQ) was calculated using the comparative 2^−ΔCt^ method (Schmittgen and Livak, 2008). Normalization was performed against the *EEF2* reference gene for each sample and subsequently related to the average gene expression of the mild samples for each gene analyzed. All TaqMan® primers and probes are listed in Table 1.

**TABLE 1:** Gene target and TaqMan® primer/probe assays.

Gene target	TaqMan® primer/probe assays
*EEF2*	Gg03339740
*TLR4*	Gg03354643
*DCN*	Gg03355063
*LUM*	Gg03325844
*BGN*	Gg07177841
*LOX*	Gg03340182
*TGF-β1*	Gg07156069
*COL1A1*	Gg07167955
*COL3A1*	Gg03325764
*SDC-1*	Gg07175697
*SDC-2*	Gg03345644
*SDC-3*	Gg03339851
*SDC-4*	Gg03370419
*MMP2*	Gg03365277
*MMP9*	Gg03338324
*TIMP2*	Gg07157666
*PDGFRβ*	Gg07165531
*ACTA2*	Gg03352404

### 2.6 Peptide synthesis, epitope mapping, and specificity testing with blocking peptides

Syndecan-1 to syndecan-4 cytoplasmic parts (mouse/chicken) were synthesized as overlapping 20-mer peptides on a cellulose membrane using a MultiPep automated peptide synthesizer, as previously described by Frank and Overwin (1996). The peptide arrays were blocked for 1 h at RT in 1% casein and thereafter overlaid with chicken syndecan-1 to syndecan-4 antibodies (1:1000) in 1% casein overnight at 4°C, washed three times for 5 min in TBS-T before incubation with anti-rabbit IgG conjugated with horseradish peroxidase (HRP) (#NA934V, Cytiva, GE Healthcare Life Sciences, MA, United States) for 1 h at RT. After further three washes in TBST-T, the signal was developed using ECL Prime (RPN 2236, GE Healthcare, IL, United States). The blocking peptides specific for each of the four chicken syndecan antibodies were synthesized to >80% purity by GenScript Corporation (United States) (syndecan-1: NGGYQKPHKQE; syndecan-2: RKPSSAAYQKAPTK; syndecan-3: KQANVTYQKPDKQE; and syndecan-4: DLGKKPIYKKAPTN).

### 2.7 Western blotting

For Western blotting, proteins were isolated from 50–100 mg of tissue samples. Protein extracts were prepared using the Precellys Lysing Kit CK28 (#P000911-LYSKO-A.0, Bertin Technologies, France) with 1 mL of Tris–EDTA lysis buffer (50 mM Tris and 10 mM EDTA, pH 8.3) supplemented with phosphatase inhibitor cocktail 2 (#P5726, Sigma-Aldrich, Merck) and AEBSF protease inhibitor (#78431, Thermo Fisher Scientific, United States). Tissue samples were homogenized two times for 20 s with a 5-s break at 6,000 rpm using the Precellys Evolution homogenizer (Bertin Technologies, France). The homogenized sample was mixed in a 1:1 ratio with 2x treatment buffer without DTT (0.125 M Tris, pH 6.8, 4% SDS, and 20% glycerol), heated for 20 min at 50°C, and centrifuged at 13,000 g for 30 min at 4°C. The supernatant, containing soluble proteins, was collected and stored at −80°C until analysis. The protein concentration was determined using DC (#5000112, Bio-Rad, CA, United States) or the Micro BCA Protein Assay Kit (#23235, Thermo Fisher Scientific, MA, United States).

Samples were prepared for Western blotting according to the NuPAGE reagent kit’s protocol (Invitrogen, Massachusetts, United States) with deviation from the official protocol of boiling samples for 5 min at 80°C. In some experiments, the samples were mixed with 4x sample buffer, containing SDS and DTT, and boiled at 95°C. The 4%–15% Criterion TG Precast Gel (#5671084, Bio-Rad) was loaded in equal amounts with 40 or 60 μg of proteins per well. Standard molecular weights used were either ECL Plex Rainbow (#RPN851E, Cytiva, GE Healthcare Life Sciences, MA, United States), Dual Xtra Standards (#1610377, Bio-Rad), Precision Plus Protein All Blue (#1610373, Bio-Rad), or Dual Color (#1610374, Bio-Rad). The gels were blotted onto PVDF membranes (#1704157, Trans-Blot Turbo Transfer Pack, Bio-Rad) using the Trans-Blot Turbo system (Bio-Rad). The PVDF membranes were blocked in 1% casein (Western Blocking Reagent, #11921681001, Sigma Merck), BSA (#805090, Norsk Labex, Norway), 1x blocking solution (Roche), or 2% ECL blocking buffer (#RPN2125, Cytiva, GE Healthcare Life Sciences, MA, United States) in TBS-T for 60 min at RT, followed by incubation with primary antibodies (Table 2) overnight at 4°C. Membranes were washed three times for 10 min each in TBS-T before being incubated with either secondary antibodies (Amersham ECL Plex Cy3 (#PA45011, anti-mouse, Cytiva, GE Healthcare Life Sciences, MA, United States) and Cy5 (#PA43009, anti-rabbit, Cytiva, GE Healthcare Life Sciences, MA, United States)) diluted at 1:2500 in TBS-T and incubated in the dark at RT for 1 h or with horseradish-peroxidase-conjugated secondary antibodies (anti-mouse IgG, NA931V, or anti-rabbit, NA934V, both from Cytiva) diluted at 1:3000 in TBS-T, followed by another three rounds of 10-min washes in TBS-T. Blots were developed using G:BOX (Syngene International Ltd., United Kingdom) for fluorescence secondary antibodies and ECL Prime (#RPN2232/2236, GE Healthcare, IL, United States) for chemiluminescence signal detection. The chemiluminescence signals were detected using Las 4000 (GE Healthcare Life Sciences, MA, United States) or Azure Biosystems (CA, United States). To compare phosphorylated and total protein, the membranes were re-probed after stripping using the Restore Western blot Stripping buffer for 5 min at RT (#21063, Thermo Fisher Scientific, MA, United States) and washed 3 × 15 min in TBS-T before blocking. Quantification of Western bands was done using ImageQuant (Cytiva, GE Healthcare Life Sciences, MA, United States). For the blocking experiment, the blocking peptides (GenScript) were pre-incubated with the respective chicken syndecan-1–4 antibodies overnight at 4°C prior to blotting of the membranes for 2 h at room temperature.

**TABLE 2:** Primary antibodies for Western blotting.

Antibody	Cat. no.	Producer	Dilution
pThr202/Tyr204-ERK1/2	#4695	Cell Signaling	1:1000
ERK1/2 total	#9272	Cell Signaling	1:1000
pSer240/244-rpS6	#2215	Cell Signaling	1:1000
rpS6	#2217	Cell Signaling	1:1000
AKT total	#9101	Cell Signaling	1:1000
pSer473-AKT	#4060	Cell Signaling	1:2000
Syndecan-1		GenScript	1:1000
Syndecan-2		GenScript	1:1000
Syndecan-3		GenScript	1:1000
Syndecan-4		GenScript	1:1000
TLR4	LSC756	LSBio	1:500
TIMP2	MBS9610370	MyBioSource	1:1000
TIMP1	sc-5538	Santa Cruz	1:200
MMP2	ab37150	Abcam	1:200
MMP9	NBP1-57940	Novus Biologicals	1:1000
Wnt7a	ab1000792		1:1000
Wnt4	ab91226		1:1000
β-catenin	#610153		1:1000
β-catenin non-phospho (active) Ser33/37/Thr41	#8814		1:1000
Dact1	#24768		1:1000

### 2.8 Zymography

Gelatinase activity was measured using gelatin zymography. Samples (approximately 300 mg) were homogenized in 2-mL tubes with ceramic beads (Precellys CK28, Bertin Technologies) and 1 mL cold buffer containing 50 mM Tris and 10 mM EDTA. Samples were kept on ice all the time. The homogenate was centrifuged, and the supernatant was collected and stored at −80°C until further analysis. Prior to electrophoresis, equal volumes of samples were mixed with the 2x Tris-Glycine sample buffer (#LC2645, Invitrogen, MA, United States). The samples were loaded on Novex® 10% Zymogram gelatin gels (#ZY00100BOX, Invitrogen, MA, United States) and run at 125 V for 100 min using the Tris-glycine running buffer (#LC2646, Invitrogen, MA, United States). The gels were washed for 30 min in the Novex™ Zymogram Renaturing Buffer (#LC2670, Invitrogen, MA, United States) and 30 min in the Novex™ Developing Buffer (#LC2671, Invitrogen, MA, United States) at RT; subsequently, they were incubated overnight at 37°C with a change to a new incubating buffer. The next day, gels were washed three times for 5 min each in distilled water, stained with SimplyBlue™ SafeStain (#LC6060, Invitrogen, MA, United States) for 1 h, and destained in distilled water overnight. Images of the gels were scanned using an Epson Perfection 4990 Photo Scanner (Epson America Inc., CA, United States). The bands were quantified using ImageQuantTL v 10.2-499 (Cytiva, GE Healthcare Life Sciences, MA, US).

### 2.9 ELISA

Competitive ELISA for heparan sulfate detection was performed in a 96-well on undiluted chicken serum collected after slaughtering, following the protocol of the heparan sulfate (MyBioSource, #MBS7217332) and syndecan-4 ELISA kits (MyBioSource, #MBS2514381). Subsequently, we determined the optical density at 450 nm using a microplate reader immediately after adding the stop solution.

### 2.10 Statistical analysis

All data of Western blots were expressed as mean ± SEM (standard error of mean, *n* = 6–8). For quantification of Western blots and zymography, ImageQuantTL v 10.2-499 (Cytiva, GE Healthcare Life Sciences, MA, US) was used with the background method of a rolling ball (radius 2). The statistical analyses of qPCR (*n* = 8) and Western blots were performed in GraphPad Prism version 8.0.1 (GraphPad Software, La Jolla, CA, United States), using a nested *t*-test for qPCR and a *t*-test with Welsh correction for Western blots. Statistical significance was considered at *p*-values <0.05 indicated in each figure. Explorative multivariate analysis by PCA was performed on all RNA-seq data and the collagens from the RNA-seq data. PCA was also applied to the NIR spectroscopic data to extract and quantify the spectral component related to WB.

## 3 Results

### 3.1 Histology classification, NIR spectroscopy, and RNA-seq analysis

**FIGURE 1:**
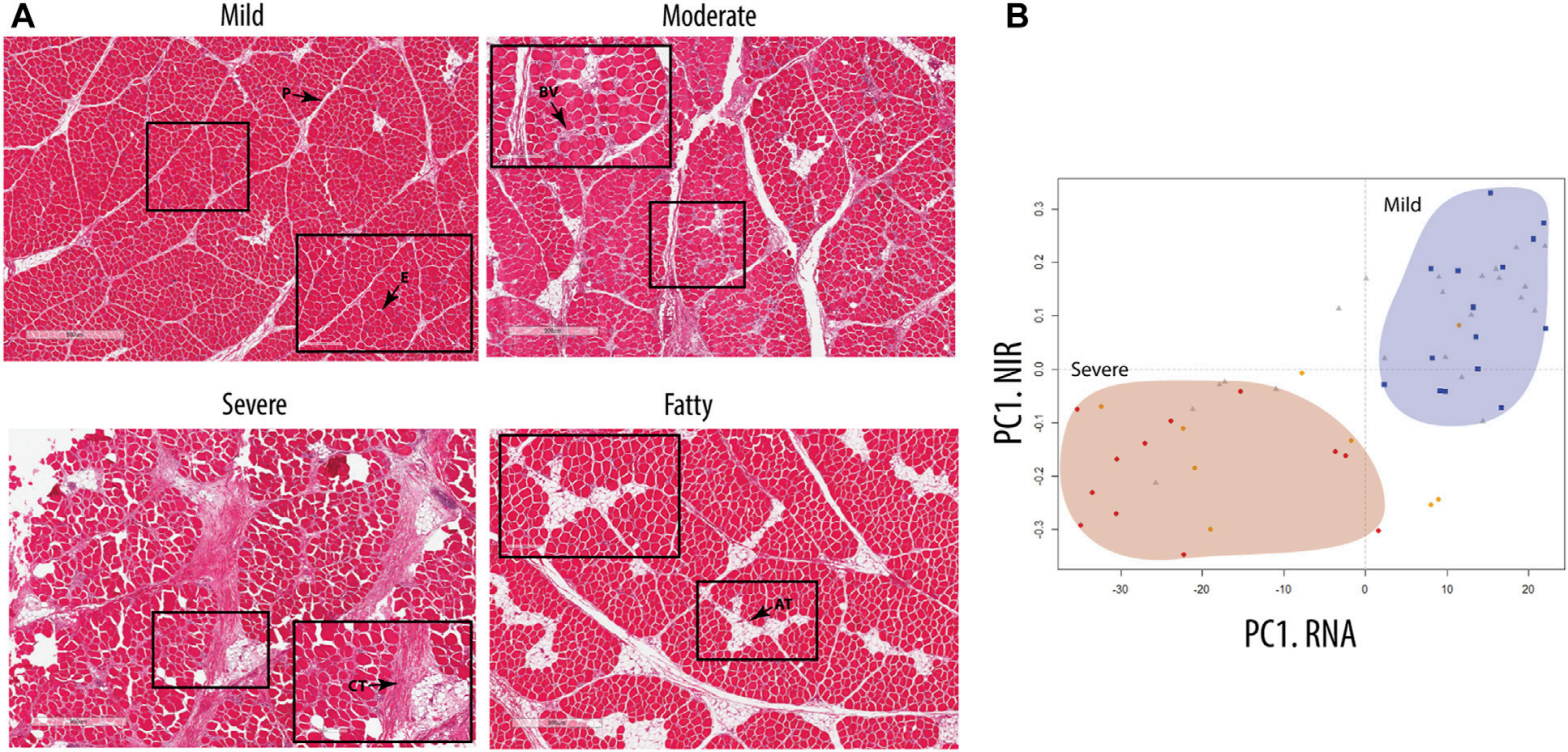
Classification of the groups. **(A)** Hematoxylin- and eosin-stained nuclei (a purplish blue) and eosin-stained extracellular matrix and cytoplasm (pink). E, epimysium; P, perimysium; CT, connective tissue; AT, adipose tissue; BV, blood vessel. **(B)** Score plot model based on RNA-seq data of chicken samples, showing group distribution comparable to data obtained from NIR spectra of cold chicken fillets measuring the amount of protein and the degree of water binding in the breast tissue. Visible separation of mild samples (blue square) and severe samples (red circle), with the moderate sample (gray triangles) distributed between them. Fatty samples (orange circle) are separated over the groups with a tendency toward the severe group.

All chicken samples (*n* = 60) were classified based on the severity of WB using H&E staining and NIR spectroscopy, grouping the samples into the following pathological categories: mild, moderate, and severe. Histology showed that the distribution of collagen-rich areas and adipose infiltrations in the perimysium varied in the WB-severe groups (Figure 1A). According to histology, a fourth group was observed, characterized by extensive adipose infiltration as well as a thinner perimysium (Figure 1A, bottom). Further investigation by RNA-seq showed a similar grouping to that achieved through NIR spectroscopy, with a clear separation between mild and severe groups (Figure 1B). The scores of the first principal components from NIR spectroscopy (*Y*-axis) and RNA-seq (*X*-axis), respectively, were combined into one plot in Figure 1B, showing a clear separation between the mild group (blue squares) and severe group (red dots), whereas the moderate group (gray triangles) was spread along the first principal component (PC). The corresponding observation by NIR revealed low water binding in the samples with severe WB compared to the mild samples. The PCA of NIR measurements also, to some extent, discriminated between samples with high- and low-fat content (data not shown), which also corresponded to samples showing low- and high-fat deposits by histology. Since the main focus of this study was to examine ECM remodeling and fibrosis, the remaining analyses were, therefore, performed on the most separated groups, the mild and severe groups, excluding the medium and fatty groups.

**FIGURE 2:**
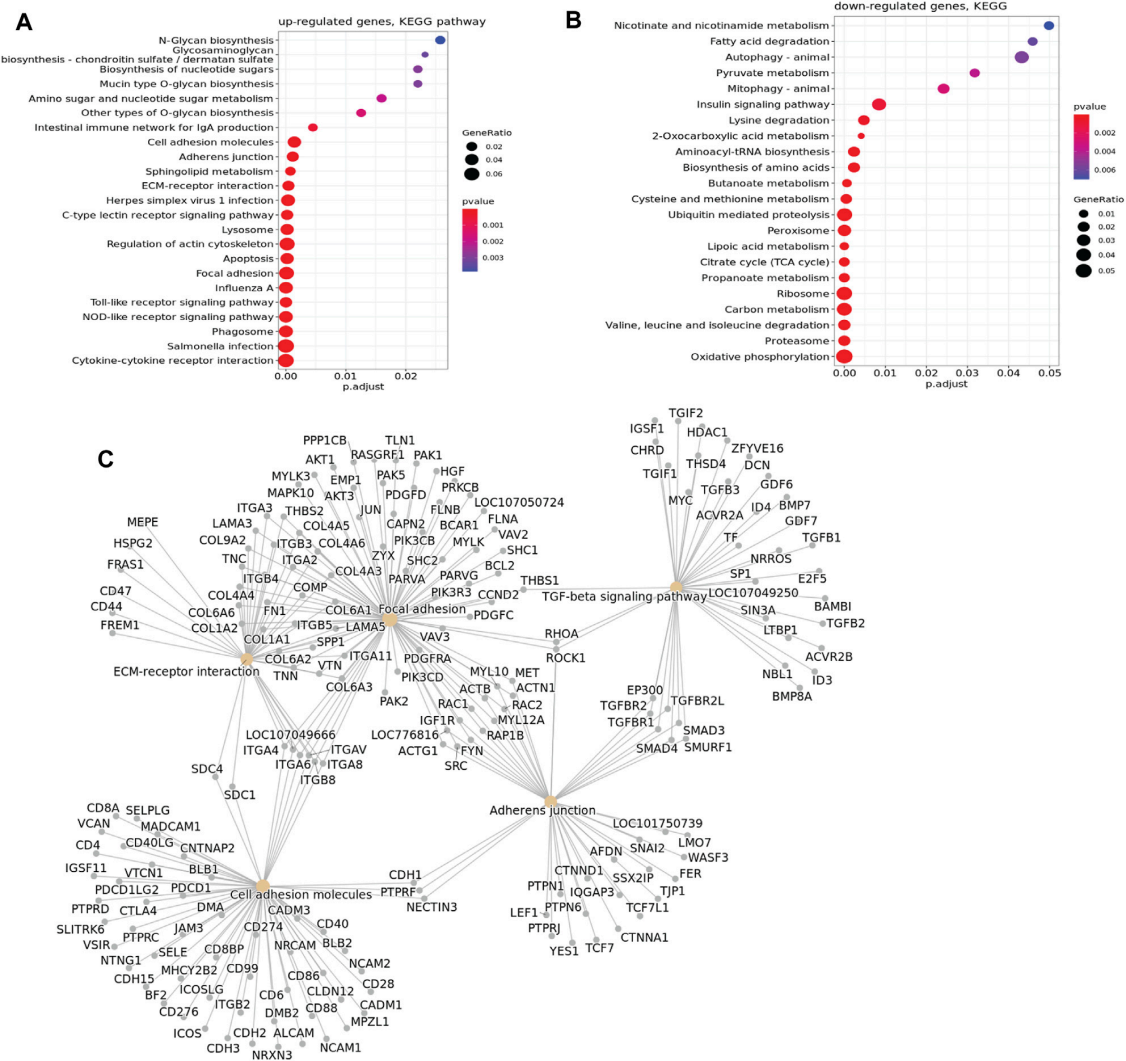
KEGG pathway enrichment bubble plot. **(A)** Upregulated genes according to KEGG pathway analysis. **(B)** Downregulated genes according to KEGG pathway analysis. In the graph, a larger dot size corresponds to more proteins in a given pathway. Additionally, a higher ratio of pathway proteins to the total proteins in the graph indicates a more significant difference in protein concentration within that pathway, suggesting a greater number of genes involved. **(C)** Cluster of upregulated genes in selected KEGG pathways associated with ECM remodeling, showing upregulation of SDC-1, SDC-4, and several other molecules studied.

The RNA-seq revealed major changes in several signaling pathways and processes between mild and severe WB cases; many of these changes are important for ECM remodeling and fibrosis. Altogether, 4,324 genes were observed to be upregulated in the severe group *versus* the mild group, whereas 4,934 genes were downregulated. The difference in the gene expression pattern between mild and moderate groups was minor, with only 21 genes upregulated and 286 genes downregulated (data not shown). Functional enrichment analysis of genes upregulated in severe cases compared with mild cases revealed molecular fingerprints of receptors and signaling pathways involved in inflammatory processes and apoptosis, glycosaminoglycan biosynthesis, ECM–receptor interactions, cell adhesion molecules, focal adhesion, and adherent junctions (Figure 2A). In contrast, downregulated genes are prevalently linked to metabolic functions such as protein and fat metabolism, especially various catabolic degradation pathways (Figure 2B). Bioinformatic cluster analysis of selected KEGG pathways connected to ECM remodeling, including pathways of cell adhesion, focal adhesion, TGF-β signaling, and adherent junctions, identified several molecules, such as *SDC-1* and *SDC-4*, together with several collagen types, integrins, *FN1* (Fibronectin 1), *TGF-β1*, *TGF-β2*, and *DCN* (decorin) (Figure 2C). In addition, RNA-seq data showed upregulation of several genes of collagen crosslinking enzymes and SLRP members, such as *LOX* and *LUM* and *FMOD* (Supplementary Table S1). Moreover, we observed increased expression of fibrillar *COL1A1* and *COL3A1* in severe samples compared to mild samples. In addition, other collagen types, including fibrillar (*COL5*), fibril-associated collagens (*COL12* and *COL16*), and network-forming collagens (*COL6*), were found to be upregulated in the severe samples (Supplementary Table S1). We also investigated the change in gene expression for inflammatory markers by RNA-seq, and we observed a significant upregulation of *TLR4*, *IL-1β*, and *IL-6* in severe compared with mild samples (Supplementary Table S1). Moreover, *TLR4* gene expression by qPCR and protein expression of splicing of TLR4 (smTLR4) were elevated in WB samples compared to mild samples (Supplementary Figure S1).

### 3.2 Structural changes and ECM

**FIGURE 3:**
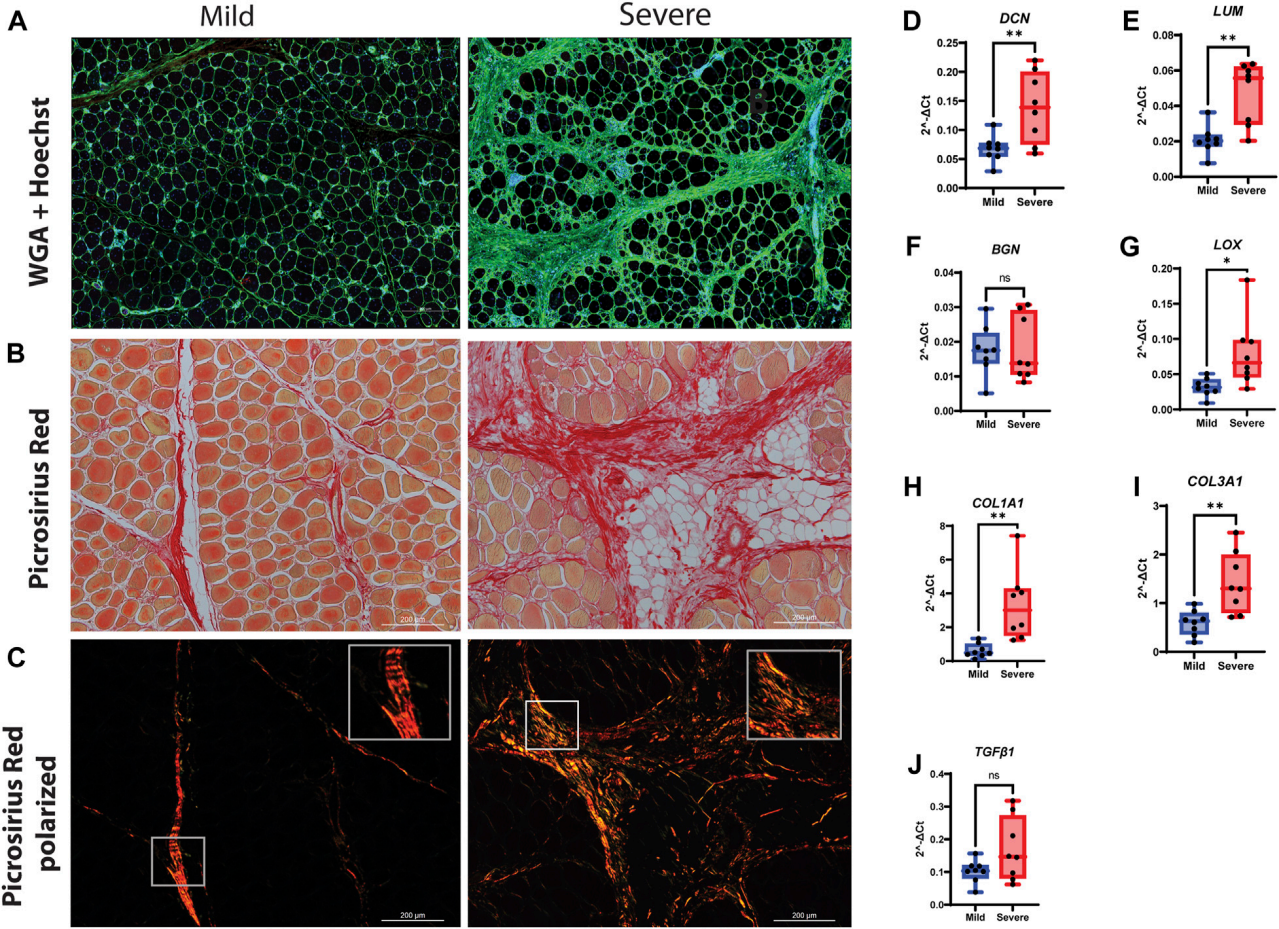
Production of connective tissue and collagen during WB. **(A)** Immunofluorescence staining of tissue by WGA shows an increased amount of connective tissue (green) in the severe group compared to the mild group. Nuclei are stained by Hoechst (Blue). **(B)** Picrosirius red visual and **(C)** polarization method. Polarized light shows the amount of new (green/yellow) and mature (red) collagen I and III and their structural differences between the two groups. **(D)** *DCN* and **(E)** *LUM* are highly upregulated in severe WB compared to the mild group. **(F)** *BGN* was shown without change. **(G)** *LOX* is upregulated in severe samples. Two types of collagens, **(H)** *COL1A1* and **(I)** *COL3A1*, are upregulated in severe samples. **(J)** *TGF-β1* shows no change between mild and severe groups. The data are presented as the fold change average relative to the mean of mild WB, ± SEM. Comparisons between the groups were analyzed using a t-test with Brown–Forsythe and Welsh correction; an asterisk indicates significant differences (ns, non-significant; *p < 0.05, **p < 0.01).

To further characterize relevant components of the extracellular matrix in more detail, we stained for sialic acid and *N*-acetylglucosaminyl residues using WGA. We observed strong staining in the endomysium surrounding each muscle fiber and in the perimysium (Figure 3A), indicating extensive ECM deposition. To investigate the structural organization and possible changes in collagen I and III organization, picrosirius red staining was used combined with light microscopy in a bright field (Figure 3B) and polarized light (Figure 3C). In the bright-field mode, the severe samples showed denser matrix structures than the mild samples. In polarized light, the perimysium in mild WB samples appeared as a compact and continuous red string, indicating that the collagen bundles were even-sized, aligned in parallels, and tightly packed, which is consistent with previous studies (Sanden et al., 2021). In contrast, samples from severely affected tissues had more yellow/orange areas, suggesting the production of new collagen in a more disordered structure (Figure 3C).

Using RT-qPCR to validate the RNA-seq data, our results showed an upregulation of the SLRPs, *DCN* and *LUM*, in severe samples compared with mild samples (Figures 3D, E). No difference in gene expression was observed for biglycan (*BGN*) (Figure 3F), while the expression level of the collagen crosslinking enzyme lysyl oxidase (*LOX*) was increased in severe WB compared to mild WB (Figure 3G). Likewise, the gene expression of *COL1A1* and *COL3A1* was increased (Figures 3H, I). The pro-fibrotic cytokine *TGF-β1* showed upregulation in severe samples when analyzed by RNA-seq analysis (Supplementary Table S1) and showed an increasing trend (not significant) when analyzed by RT-qPCR (Figure 3J). No differences in the gene expression of myofibroblast markers, *PDGFRβ* and *ACTA2*, between the groups were observed (Supplementary Figures S2A, B). Furthermore, no differences in α-smooth muscle actin (α-SMA) protein expression were observed when analyzed by Western blotting (Supplementary Figure S2C).

### 3.3 Syndecan expression

**FIGURE 4:**
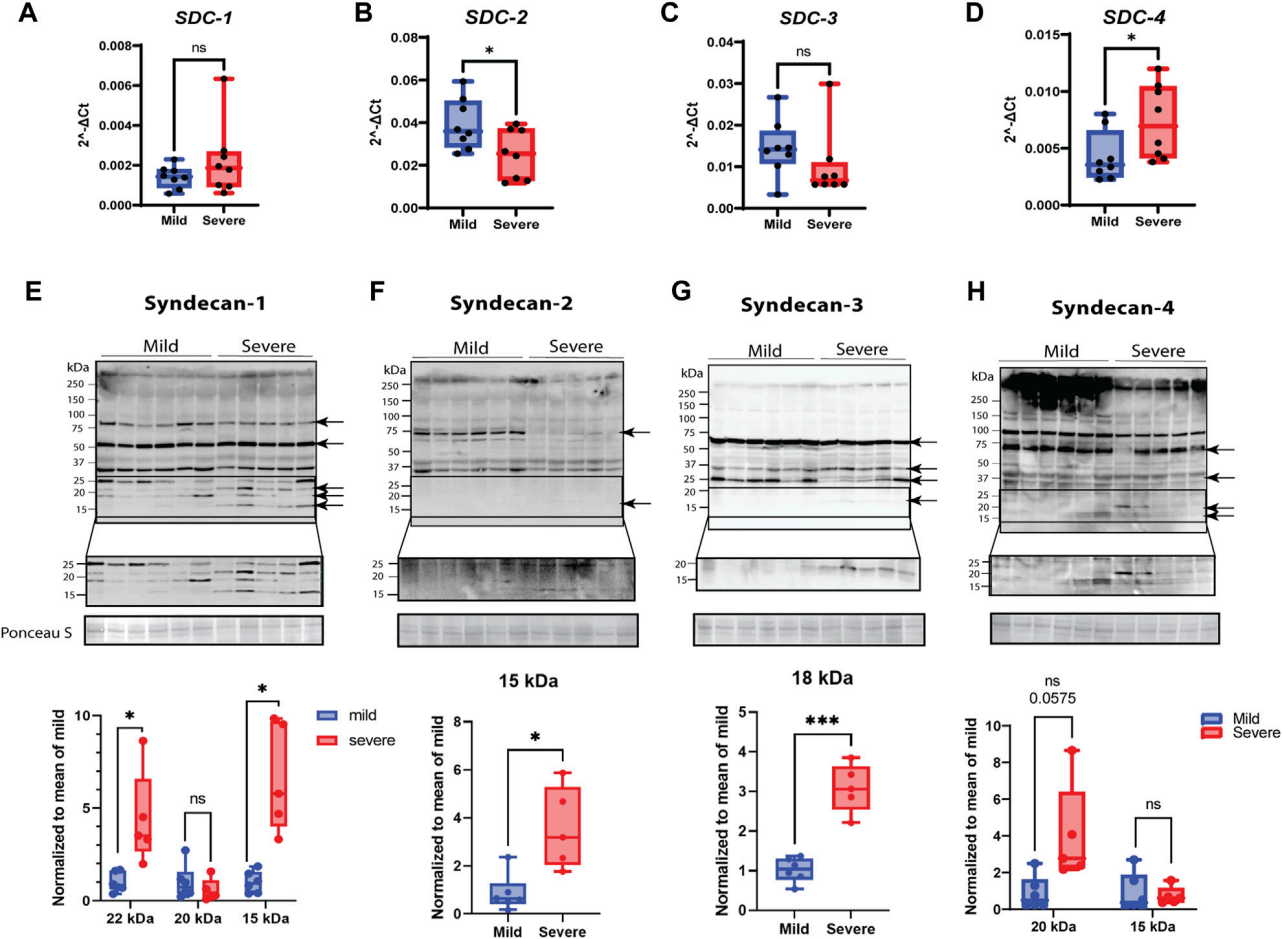
Gene and protein expression of syndecans and their shedding. **(A–D)** Gene expression monitored by RT-qPCR. Gene expression of **(A)** *SDC-1* by qPCR shows variety between samples. **(B)** *SDC-2* qPCR data shows downregulation in severe groups, and **(C)** *SDC-3* gene expression was insignificant between groups. **(D)** *SDC-4* qPCR data show significantly elevated gene expression in severe samples. **(E–H)** Protein expression of syndecans and their shedding. The specific bands are highlighted by arrows (confirmed by blocking experiments in Supplementary Figure S4). The lower part of the pictures shows increased signal intensity of the syndecan C-terminal domain after shedding. **(E)** Syndecan-1 shows extensive shedding between 15 and 25 kDa in severe samples. **(F)** Syndecan-2 is mainly present as a protein band of 75 kDa in mild samples and shows a weak, significant 15-kDa shed fragment in the severe group. **(G)** A shed fragment of 15 kDa of syndecan-3 was detected in severe samples. **(H)** Syndecan-4 shows a decreased amount of protein in the higher-molecular-weight area (>250 kDa) and shows several increased shed fragments of approximately 20 kDa (tendency). The qPCR data are presented as the fold change average relative to the mean of mild WB, ± SEM. Comparisons between the groups were analyzed using a nested t-test with Brown–Forsythe and Welsh correction (, *p < 0.05). Shedding of syndecans was normalized to the mean of the mild group and analyzed using an unpaired t-test with Brown–Forsythe and Welsh correction; asterisks indicate significant differences (ns, non-significant; *p < 0.05, ***p < 0.001).

Since transcriptomic analysis demonstrated that genes involved in cell adhesion were upregulated in muscles from severely affected animals (Figure 2A, KEGG pathway analysis, Supplementary Table S1), we sought to inspect in more detail key players of cell adhesion, with a focus on the syndecan family. RT-qPCR analysis of relative gene expression of *SDC-1* and *-3* showed no significant changes between the mild and severe groups (Figures 4A, C). In contrast, *SDC-2* was downregulated in severely affected muscles compared with mildly affected muscles (Figure 4B), while *SDC-4* was upregulated (Figure 4D).

When examining protein expression by Western blotting using custom-made chicken-specific antibodies generated against the cytoplasmic domain of the four syndecans (Supplementary Figure S3, epitope mapping), we observed numerous protein bands (Figures 4E–H). To determine the bands specific to syndecan-1–4, we performed blocking experiments using specific peptides containing the epitope for each of the four developed chicken syndecan antibodies (Supplementary Figure S4). The syndecan-1–4-specific bands, as verified by the peptide blocking experiments, ranged from 15 to 90 (syndecan-1), 15 to 75 (syndecan-2), 15 to 60 (syndecan-3), and 15 to 250 (syndecan-4) kDa (Figures 4E–H, indicated with arrows). It is possible that these higher-molecular-weight bands are SDS-resistant homo- or perhaps hetero-oligomers of syndecans. We could observe similar levels of a possible syndecan-1 oligomer of 80 kDa in the mild and severe groups; however, a possible syndecan-2 oligomer of 70 kDa was only observed in the mildly affected group. For syndecan-3, we observed a possible oligomer of 60 kDa at a similar level in both groups. The very-high-molecular-weight (HMW) bands observed in all four Western blots (>250 kDa) were prominently reduced for syndecan-4 in the severe group vs. mild group. Interestingly, we could also observe a distinct pattern of smaller fragments (<25 kDa) for all the syndecans in the severe group (Figures 4E–H, see increased contrast of the lowermost panels). These bands are likely the remaining transmembrane and cytoplasmic parts of the syndecans after shedding. Syndecan-1 exhibited several smaller fragments, probably reflecting a strong degree of shedding of ectodomains, but smaller fragments were also observed for syndecan-2, -3, and -4 (quantified in the lower panels in Figures 4E–H). We were unable to detect shed syndecan-4 in blood serum using commercial ELISA kits (data not shown); however, ELISA for HS chains demonstrated significantly reduced concentrations of HS in severe samples compared to mild samples (Supplementary Figure S4). Notably, the relative gene expression of heparanase was upregulated in severe WB (Supplementary Table S1).

### 3.4 MMP activity

**FIGURE 5:**
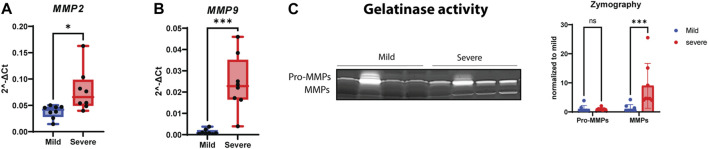
Increasing activity of MMPs in WB. Gene expressions of **(A)** *MMP2* and **(B)** *MMP9* show significant upregulation in severe WB. **(C)** A representative gelatin zymogram for measurement of pro-MMP and MMP activity in mild and severe WB samples and the quantified activity of MMP activity (*n* = 8 in each group). Comparisons between the groups (mean ± SEM, *n* = 8) were analyzed using a t-test with Brown–Forsythe and Welsh correction; asterisks indicate significant differences (ns, non-significant; *p < 0.05, **p < 0.01, ***p < 0.001).

To investigate whether MMPs were involved in WB pathogenesis, we analyzed *MMP2* and *MMP9* gene expression using RT-qPCR, assessed protein expression using Western blotting, and examined their gelatinase activity. Although both *MMP2* and *MMP9* gene expression levels were upregulated in severe samples (Figures 5A, B), their protein levels were downregulated in severe compared to mild samples (Supplementary Figures S5A, B). In contrast, the MMPs had increased activity in severe WB samples (Figure 5C). Notably, the pro-form of MMPs was unchanged between groups (Figure 5C). The analysis of protein expression of MMP inhibitors, specifically, TIMP-1 and TIMP-2, through Western blotting showed no differences in their protein levels (Supplementary Figures S5C, D); however, the *TIMP2* gene level showed a tendency to be upregulated in severe WB (Supplementary Figure S5E).

### 3.5 MAPK, AKT, and Wnt signaling

**FIGURE 6:**
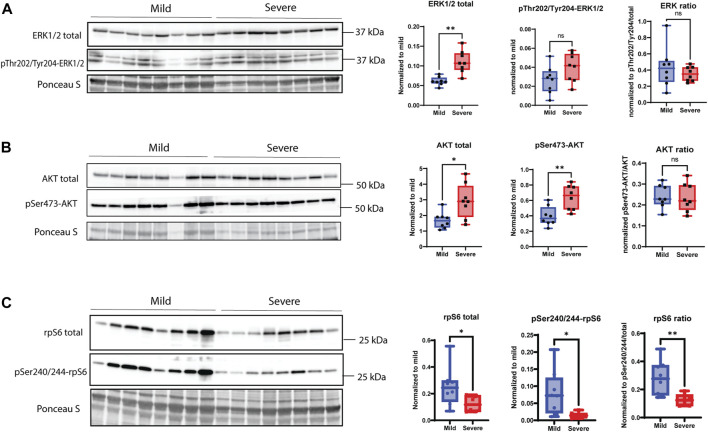
MAPK signaling pathway. **(A)** Total ERK1/2 is significantly upregulated in severe WB samples; however, pThr202/pTyr204-ERK1/2 shows no change between groups, and the overall ratio is not significant between mild and severe samples. **(B)** pSer473-AKT and total AKT are significantly increased in severe WB samples, but the overall ratio is not significant between groups. **(C)** pSer240/244-rpS6 and total rpS6 are downregulated in severe samples, and the total ratio is decreased in mild WB samples. Blots for phosphorylated version of proteins were reused for the detection of total version of proteins. Comparisons between the groups (mean ± SEM, *n* = 8) were analyzed using a t-test with Brown–Forsythe and Welsh correction; asterisks indicate significant differences (ns, non-significant; *p < 0.05, **p < 0.01).

According to the RNA-seq data (Supplementary Table S1), genes belonging to signaling pathways responsible for ECM remodeling, cell receptor signaling, and focal adhesions were significantly upregulated in WB pathogenesis. Increased levels of the total extracellular signal-regulated kinase 1/2 (ERK1/2) and its phosphorylated version (Thr202/Tyr204, tendency), which are parts of the MAPK signaling pathway, showed an increase in severe WB myopathy (Figure 6A). Increased levels were also observed for protein kinase B (AKT) and pSer473-AKT (Figure 6B). Surprisingly, ribosomal protein S6 (rpS6) and the phosphorylation of its serine residues 240 and 244 (pSer240/244), which is a downstream target of the AKT/mTOR signaling pathway, were both downregulated in severe samples (Figure 6C).

**FIGURE 7:**
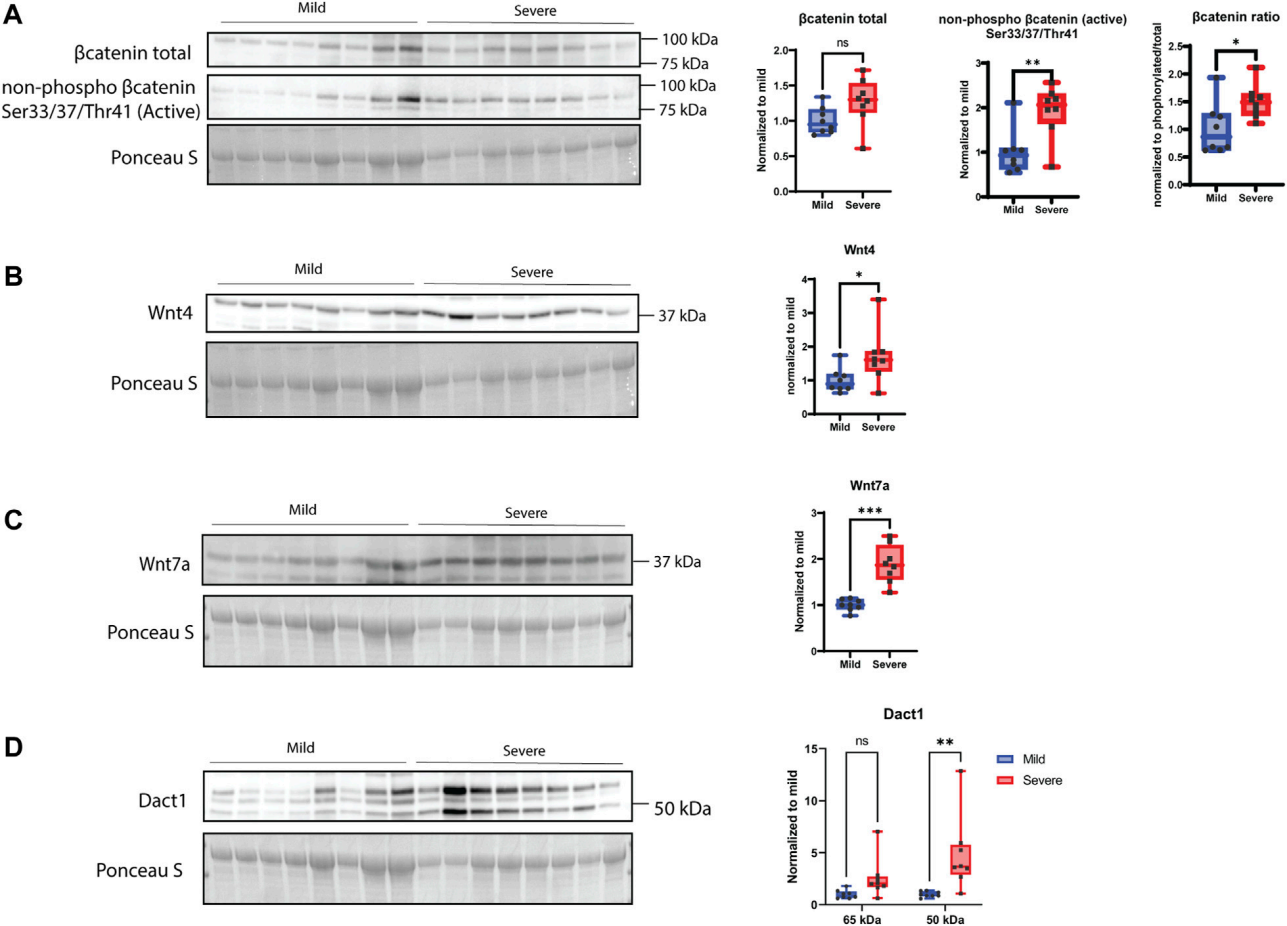
Wnt signaling. **(A)** β-catenin shows no significant difference between mild and severe groups, and its active non-phosphorylated version is upregulated in severe WB samples. The ratio is increased in severe WB. **(B)** Wnt4 and **(C)** Wnt7 are significantly upregulated in severe WB samples. **(D)** Dact1 was mainly detected as two bands (50 kDa and 65 kDa) and shows an increase of the 65 kDa band in severe samples. Blot was reused for both versions of β-catenin and Wnt4, and another blot was used for Wnt7 and Dact1. Comparisons between the groups (mean ± SEM, *n* = 8) were analyzed using a t-test with Brown–Forsythe and Welsh correction; asterisks indicate significant differences (ns, non-significant; *p < 0.05, **p < 0.01, ***p < 0.001).

When examining players of the Wnt signaling pathway, including members of the canonical and non-canonical pathways, we could observe an increase in the active form of *ß*-catenin and a tendency for increased production of total *ß*-catenin in severe WB samples (Figure 7A). Moreover, Wnt-4 and Wnt-7a proteins were also found to be upregulated in severe cases of WB (Figures 7B, C). Furthermore, Dact1 (disheveled binding antagonist of *ß*-catenin 1) of 50 kDa showed no significant change between groups, whereas a 65-kDa form was found to be elevated in the severe group (Figure 7D).

## 4 Discussion

### 4.1 Extracellular matrix undergoes extensive remodeling during wooden breast pathogenesis

In the present study, we investigated the histological and molecular profiles of fibrosis, as this is a hallmark of WB pathogenesis. To classify the severity of WB, we used NIR, an established method for the measurement of WB severity (Wold et al., 2017; Wold et al., 2019), together with the histology of connective tissue distribution. Histological analyses demonstrated that all the chickens had some signs of WB; hence, they were classified as mild, moderate, and severe. Previously, NIR spectral information displayed up to 97.5% accuracy in the classification of wooden breast samples (Geronimo et al., 2019). Consistently, in our NIR analysis, the severely affected WB samples were easily separated from the mild samples, while the moderate samples were identified both within and between the mild and severe groups. Notably, RNA-seq data and histology analyses resulted in a similar classification to the NIR analysis, separating the severe group with heavy fibrosis from the mild group with minimal signs of fibrosis.

The severe WB group showed a huge variation in gene expression levels compared with mild WB. Our RNA-seq data identified glycosaminoglycan biosynthesis, ECM–receptor interactions, focal adhesions, cell adhesion molecules, toll-like receptor signaling pathways, and apoptosis as the most prominent upregulated genes in severe WB. Syndecan family members, especially syndecan-4, were found upregulated in severe WB with increased shedding of their ectodomains. In line with previous reports (Papah et al., 2018; Brothers et al., 2019; Malila et al., 2021), ECM components such as SLRPs, different collagen types, and the collagen crosslinking enzyme *LOX* were upregulated. The increased *LOX* level probably resulted from the increased inflammatory response observed in severe WB. Inflammation has been shown to induce LOX and lysyl hydroxylase expression *via* the hypoxia-inducible factor 1α (Hofbauer et al., 2003). Moreover, disruption of focal adhesion signaling has been reported to be accompanied by defective ECM, insufficient angiogenesis, poorer tissue oxygenation, impaired wound healing, aberrant depositions of ECM proteins, and, ultimately, fibrosis in mice (Koivisto et al., 2013).

Fibrosis-induced tissue hardness is affected by both the extent and structure of fibrillar collagens (Xing et al., 2020). In our study, we observed both increased collagen depositions and rearrangement of collagen fibrillary structure in severe WB, reflecting extensive ECM remodeling, in line with previous findings by us and others (Velleman and Clark, 2015; Griffin et al., 2018; Praud et al., 2020; Sanden et al., 2021). Fibrillar collagen types I and III are the dominant collagen types in muscle (Deshmukh et al., 2016), and our gene expression analysis demonstrated the upregulation of both types in severe WB. In addition, several other collagen types, such as fibrillar (collagen type V), fibril-associated collagens (collagen types XII and XVI), and network-forming collagens (collagen VI), were upregulated. Fibrillar collagens can be cross-linked with other ECM molecules, such as fibronectin (upregulated in our dataset), to form stable networks that provide mechanical support to the tissue. Fibrillar collagens are also crosslinked by LOX. LOX contributes to increasing collagen fibril diameter and tissue hardness (Xing et al., 2021a). SLRPs, including LUM, BGN, and DCN, are all crosslinking regulators and are shown to play a role in ECM remodeling and fibrosis in different mouse models of muscular and cardiovascular diseases (Mohammadzadeh et al., 2019). Only DCN has previously been investigated in WB in relation to collagen organization and crosslinking (Velleman et al., 2017; Velleman, 2020). DCN binds to collagens and TGF-β, and its level has been found to correlate with increased fibrosis in different muscular dystrophies (Zanotti et al., 2005). Our study identified the upregulation of both *LUM* and *DCN* in severe WB samples, and both expression levels were found to be similar to those previously reported in different muscle dystrophies (Zanotti et al., 2005; Velleman, 2015; Velleman et al., 2017; Xing et al., 2020). LUM is known to play a crucial role in maintaining the integrity of cardiac ECM and is involved in tissue inflammation by regulating immune cell recruitment in different mouse models (Wu et al., 2007; Mohammadzadeh et al., 2019). On the other hand, BGN, which is an important pro-inflammatory factor acting as an endogenous ligand of TLR-4, has been found to be highly upregulated in different types of fibrosis (Schaefer et al., 2005). However, no changes were observed in the *BGN* gene expression level in our study of WB myopathy. Taken together, the upregulation of *DCN* and *LUM* in severe WB, along with the upregulation of TLR signaling pathways, suggests an involvement of DCN and LUM, but not BGN, in the inflammatory responses of WB.

### 4.2 Syndecans as key players in WB pathogenesis

Syndecans play a major role in focal adhesion regulation, as well as in ECM–receptor interactions and cell adhesion regulation (Couchman, 2010)—all of which constitute some of the most upregulated genes observed in our RNA-seq data sets. To the best of our knowledge, expression and shedding levels of the syndecan family in WB myopathy have not been previously investigated. The altered syndecan expression and shedding levels support the notion that these cell surface receptors are involved in WB skeletal myopathy, adding to their importance as skeletal muscle regulators and molecular players in ECM-tissue homeostasis. Syndecans and their role in fibrosis have primarily been studied in relation to cancer (Cheng et al., 2016), cardiac (Lunde et al., 2016; Herum et al., 2020), and lung fibrosis (Parimon et al., 2019) and skeletal muscle aging in mice (Pisconti et al., 2016). The upregulation of *SDC-1* (tendency) and *SDC-4* gene expression in severe WB, coupled with the downregulation of *SDC-2* gene expression, suggests different and specific roles for the different syndecans. In tissue regeneration, syndecan-2 plays a major role in vascularization, regulating cell adhesion, and promoting spreading (Noguer et al., 2009). In line with the reduced expression of *SDC-2*, reduced vascularization has been demonstrated in WB (Luque et al., 1995), and it is suggested to induce muscle cell necrosis and tissue damage (Reine et al., 2014; Velleman and Clark, 2015). Syndecan-1 regulates cardiac fibroblast physiology through TGF-β signaling and is revealed as a pro-fibrotic molecule (Schellings et al., 2010). Syndecan-4 plays a major role in focal adhesions and migration of fibroblasts (Li and Chaikof, 2002; Herum et al., 2013) and is involved in cardiac myofibroblast differentiation and collagen production (Herum et al., 2015). Consistent with a role for syndecan-4 in fibrosis, we have previously observed increased gene expression levels of *DCN*, *COL1A1*, *COL3A1*, *FMOD*, *BGN*, and *LOX* in skeletal muscle (*tibialis anterior*) from *SDC-4* KO mice (Rønning et al., 2020). Moreover, cardiac syndecan-4 expression is upregulated by pro-inflammatory stimuli such as TNFα and IL-1β (Strand et al., 2013a). Although we detected elevated gene expression levels of *IL-1β*, *IL-6*, and *TGF-β1* in severe WB, no differences in the inflammation markers, *VCAM1* and *IL-10*, were observed. This observation can be explained by the fact that inflammation markers are time-sensitive and depend on whether the injury is acute or chronic. Additionally, we studied α-smooth muscle actin (α-SMA and *ACTA*), which is a marker for myofibroblast differentiation and linked to syndecan-4 function (Herum et al., 2015). To our surprise, we did not detect any differences in α-SMA, neither at the gene nor protein level. In addition, the myofibroblast marker *PDGFR* was unchanged. These observations are in line with previous reports showing that α-SMA is not a functional marker of fibrogenic cells in skeletal muscle fibrosis associated with muscular dystrophy (Zhao et al., 2018). Notably, since endothelial cells also express α-SMA and reduced angiogenesis and vascular endothelial dysfunction are associated with WB (Abasht et al., 2021), a potential increase in α-SMA expression by fibrogenic cells might be zeroed out by lower α-SMA expression from endothelial cells.

Moreover, our study indicated altered oligomerization patterns of the syndecans in relation to WB pathogenesis. Syndecans form non-covalently linked homodimers through a highly conserved transmembrane domain, which contains the ubiquitously conserved GxxxG motif (Lambaerts et al., 2009). The syndecan family is also reported to have the ability to heterodimerize (Choi et al., 2015). Even in the presence of a strong anionic detergent, like sodium dodecyl sulfate (SDS), the syndecans can form dimers, a property known as SDS-resistant dimerization (Choi et al., 2015). Homo- and hetero-oligomerization properties appear essential for the function of syndecan-2 and syndecan-4 as cytoskeletal organizers and mediators for migration and adhesion (Choi et al., 2005; Choi et al., 2008; Choi et al., 2015).

The shedding of the syndecan ectodomain, containing the HS GAG chains, is a mechanism through which cell-bound HS is turned into a soluble effector molecule; this process may affect the inflammatory process (Strand et al., 2013b) and could cause fibrosis. In our study, we investigated the shedding of the syndecans using specific antibodies directed against the cytoplasmic part (C-terminus) of all four chicken syndecans. The exact shedding sites in chickens and the amounts and types of GAGs attached to different chicken syndecans are unknown. Studies have shown that the shedding of syndecan core proteins can be accelerated by pathological events and various stimuli, leading to increased expression of pro-inflammatory molecules and NF-κβ activation (Lambaerts et al., 2009). Our data using specific antibodies against the syndecan-1 to -4 cytoplasmic parts identified smaller syndecan fragments of 15–35 kDa, suggesting the shedding sites to be near the transmembrane domain and likely also within the N-terminus. Increased shedding of syndecan-4 was observed in severe WB, and consistently, a decreased amount of HMW syndecan-4 on the top of the SDS/PAGE gel, probably corresponding to the intact proteoglycan form, was also observed. The short cytoplasmic tail of syndecans regulates signaling, while their extracellular part, modified with glycosaminoglycan chains, binds to a range of extracellular matrix molecules involved in fibrosis and immune cell adhesion proteins (Lunde et al., 2016). The detailed investigation of GAG-attachment and their sulfation pattern was not the aim of this study. However, it is worth noting that the functions of syndecans depend largely on the sulfation level and pattern of the heparan/chondroitin sulfate chains, the rate of ectodomain shedding, the solubility of the ectodomains, and the different partner molecules binding to the ectodomain (Gopal, 2020). Interestingly, investigation of HS chain concentration in chicken serum showed degradation of HS in severe WB samples compared to mild samples. Consistently, we observed significant upregulation of gene expression of *HPSE* (heparanase) in WB samples (Supplementary Table S1). This is in line with data from Ozkok et al. (2018) who found that increased inflammation is associated with elevated heparanase activity, which might cause degradation of HS chains attached to soluble ectodomains in serum. Elevated shedding of syndecans is mediated by MMPs (Manon-Jensen et al., 2013), and in our study, we found that both *MMP2* and *MMP9* were increased at the transcript levels in severe WB samples, along with increased gelatinase activity. The upregulation of MMPs correlates with the inflammatory process in muscular dystrophies and inflammatory myopathies, suggesting a potential contribution to muscle regeneration (Kherif et al., 1999; Miyazaki et al., 2011). TIMPs function exclusively as endogenous inhibitors of MMPs, thereby modulating ECM degradation and remodeling (Alameddine and Morgan, 2016). Thus, the balance between MMP and TIMP levels modulates ECM homeostasis. Perturbation of this balance occurs in various physiological and pathological remodeling situations, including different muscular dystrophies, neuromuscular diseases, and inflammatory myopathies (Alameddine, 2012). In this study, RNA-seq analysis identified increased levels of *TIMP2* in severe WB, and there was a tendency of increase observed by qPCR. However, no changes in TIMP2 were observed at the protein level.

### 4.3 Signaling mechanisms in wooden breast fibrosis

Syndecans are involved in several signaling pathways, including ERK/MAPK (Hara et al., 2021), AKT/mTOR/S6K1, and Wnt signaling (Luyten et al., 2008; Jones et al., 2020; Hara et al., 2021; Johnson de Sousa Brito et al., 2021; Jones et al., 2022); however, none of them have previously been investigated in the context of WB myopathy.

The ERK1/2 signaling cascade is a crucial pathway that integrates extracellular signals from cell surface receptors to regulate gene expression and multiple cellular proteins. Ligand binding activates several intracellular proteins, including Ras, Raf, MEK, and, finally, ERK1/2, through a cascade of phosphorylation events (Foglia et al., 2019), affecting cell proliferation, differentiation, adhesion, migration, and survival (Foglia et al., 2019). Our results showed upregulation of total ERK1/2 and its phosphorylated pThr202/Tyr204 form in severe WB. We also found elevated amounts of AKT and pSer473-AKT, which are involved in muscle growth and hypertrophy (Lai et al., 2004). In contrast, phosphorylation of the downstream target Ser240/244-rpS6 was downregulated in severe WB. Previous studies have shown that ablation of syndecan-4 leads to the activation of the AKT/mTOR/S6K1 pathway in skeletal and cardiac muscle from female mice (Rønning et al., 2020; Støle et al., 2022). On the contrary, the ablation of syndecan-4 in endothelial cells is reported to result in decreased AKT activation (Partovian et al., 2008). In contrast, we observed upregulation of both syndecan-4 and AKT activity in severe WB. The disparities in results might be explained by differences in species, tissue, or perhaps sex.

It has already been shown that Wnt signaling is essential for normal heart valve development during chicken embryogenesis (Brade et al., 2006), and it has several times been linked to skeletal muscle hypertrophy (Armstrong and Esser, 2005; Steelman et al., 2006). We found alterations in both canonical and non-canonical Wnt signaling in WB myopathy, both at the gene and protein levels. In particular, active *ß*-catenin, Dact1, Wnt-4, and Wnt-7a were all upregulated in severe WB. Furthermore, RNA-seq data showed alterations in several genes involved in Wnt signaling, such as *APC*, *DACT1*, *DKK2*, *DVL2*, and *WNT4* **(** Supplementary Table S1 **)**, emphasizing an important role for Wnt signaling in WB myopathy.

## 5 Conclusion

**FIGURE 8:**
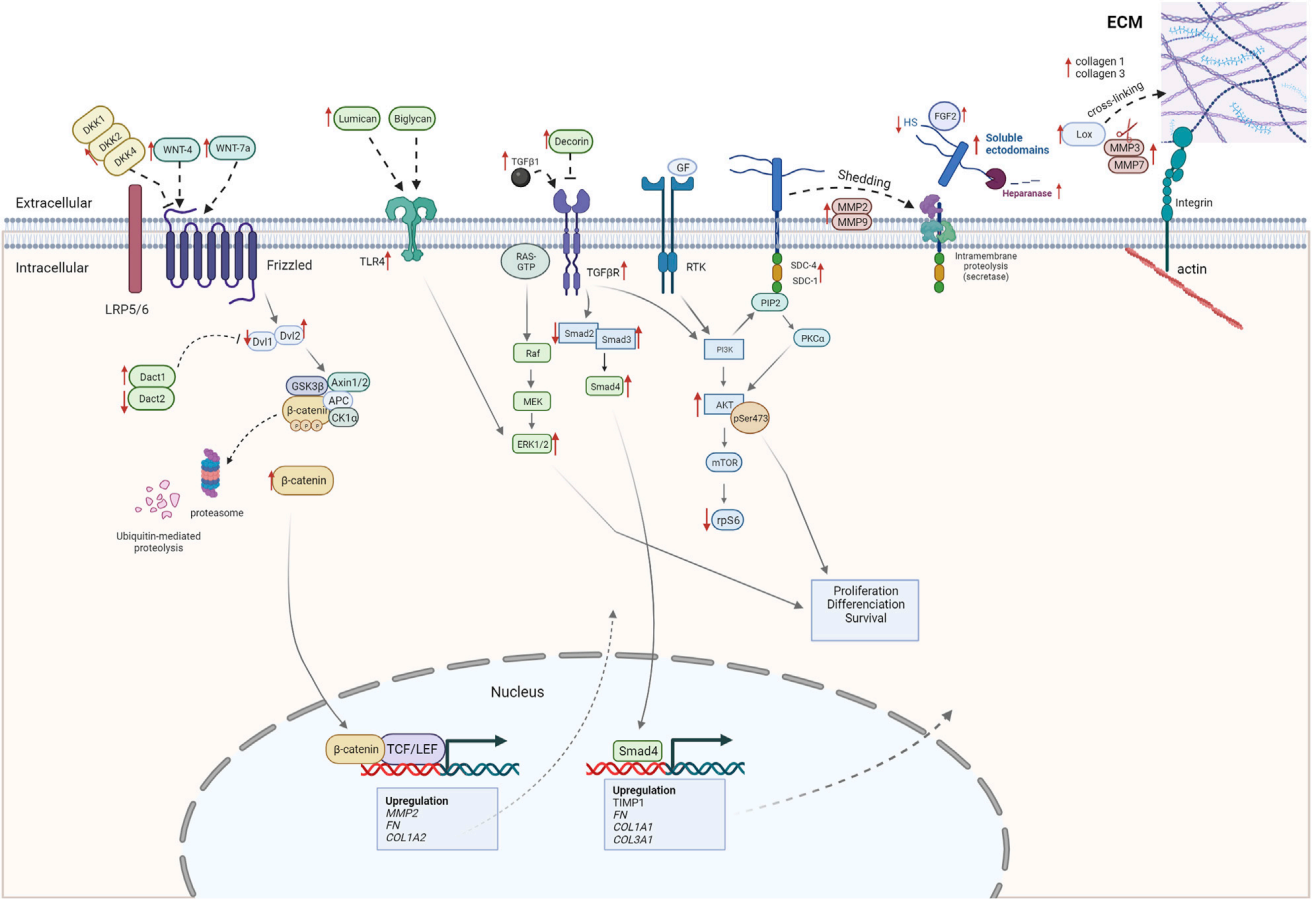
Alterations observed in severe WB myopathy (this study). The figure shows alterations observed in severe WB myopathy by RNA-seq, RT-qPCR, and Western blotting.

In conclusion, our study has focused on characterizing WB myopathy and its ECM remodeling, with a special emphasis on the syndecan family and signaling pathways involved in fibrosis. A summary of our findings obtained from RNA-seq, RT-qPCR, and Western blotting analyses is depicted in Figure 8. In brief, several collagens, SLRPs, LOX, expression of syndecan-4, shedding of all the syndecans, MMPs, heparanase activity, TLR4, and ERK/MAPK, AKT, and Wnt signaling pathways all appeared to be increased in severe WB (Figure 8). Further study of the mechanisms underlying WB myopathy is warranted.

## Data Availability

The original contributions presented in the study are publicly available. This data can be found here: https://www.ebi.ac.uk/ena/browser/view/PRJEB66500.
