# Supplementary material for: Characterization of wooden breast myopathy: a focus on syndecans and ECM remodeling
Source: Front Physiol. 2023 Dec 5;14:1301804. doi: 10.3389/fphys.2023.1301804 (PMC10737271; doi:10.3389/fphys.2023.1301804)

Supplementary data

Characterization of Wooden Breast Myopathy: A Focus on Syndecans and ECM Remodeling

Lucie Pejšková[1*], Sissel Beate Rønning[1], Matthew P. Kent[2], Nina Therese Solberg[1], Vibeke Høst[1], To Thu-Hien[2], Jens Petter Wold[1], Marianne Lunde[3], Ellen Mosleth[1], Addolorata Pisconti[4], Svein Olav Kolset[5], Cathrine Rein Carlson[3], Mona Elisabeth Pedersen[1].

[1] Raw Materials and Optimization, Nofima AS, Ås, Norway

[2] Center for Integrative Genetics, Department of Animal and Aquacultural Sciences, Faculty of Biosciences (BIOVIT), Norwegian University of Life Sciences (NMBU), Ås, Norway

[3] Institute for Experimental Medical Research, Oslo University Hospital and University of Oslo, Oslo, Norway

[4] Department of Biochemistry and Cell Biology, SUNY Stony Brook, NY, USA

[5] Department of Nutrition, Institute of Basic Medical Science, University of Oslo, Oslo, Norway

*** Correspondence:** lucie.pejskova@nofima.no

Keywords: Wooden breast, myopathy, syndecans, ECM, broiler chicken

Running title: Syndecans and remodeling in WB

Figure S1:

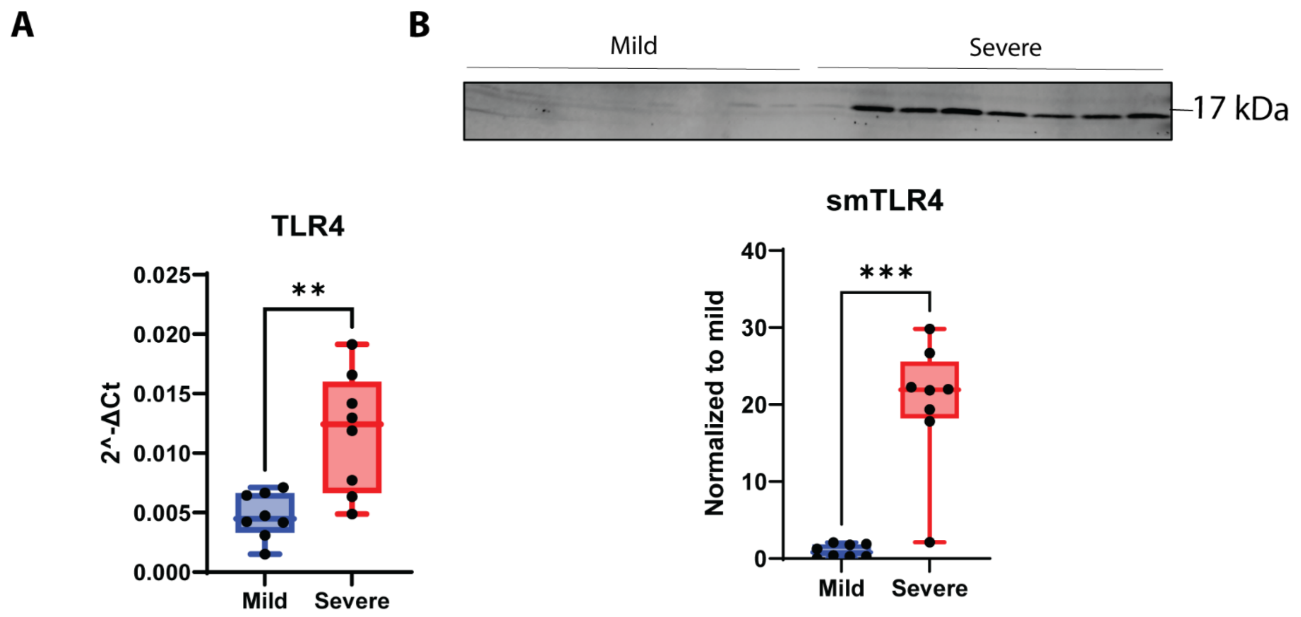

Figure S1. Toll-like receptor 4 is elevated in severe WB. (A) Bars show relative gene expression in mild and severe WB samples for the inflammation marker, *TLR4*. The data are presented as the fold change average relative to mean of mild WB, ± SEM, n=8. Comparisons between the groups were analyzed using nested t-test with Brown-Forsythe and Welsh correction (**$p < 0.01$). **(B)** A representative western blot showing a 17 kDa splicing variant of TLR4 in severely affected WB samples. Tissue lysates were subjected to western blotting using antibodies to TLR4. Western blots were quantified using ImageQuant. Comparisons between the groups (mean ± SEM, n=8) were analyzed using t-test with Brown-Forsythe and Welsh correction (** $p \leq 0.01$, *** $p \leq 0.001$).

Figure S2:

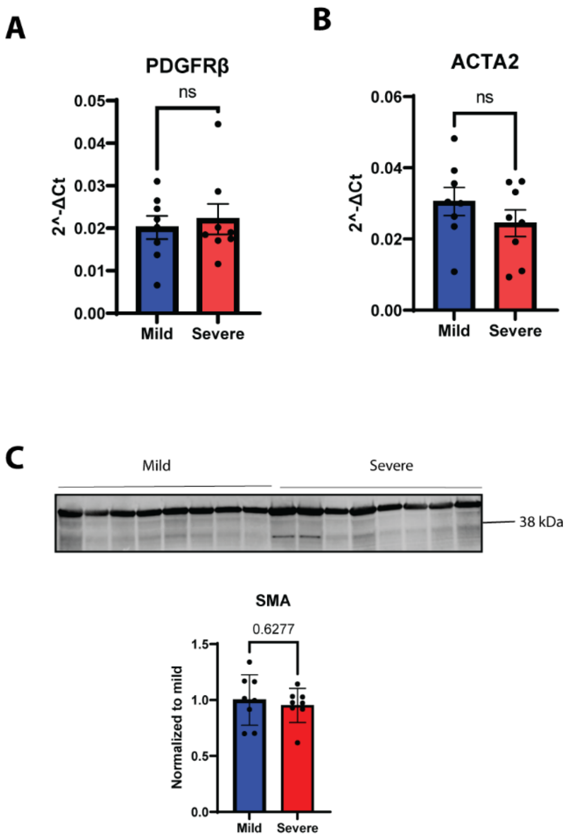

Figure S2.Gene expression of **(A)** *PDGFRβ* and **(B)** *ACTA2* monitored by RT-qPCR shows no changes between mild and severe groups. The qPCR data are presented as the fold change average relative to mean of mild WB, (n=8) ± SEM. **(C)** A representative western blot showing SMA in severely affected WB samples. Comparisons between the groups (mean ± SEM, n=8) were analyzed using t-test with Brown-Forsythe and Welsh correction (ns $p > 0.05$).

Figure S3:

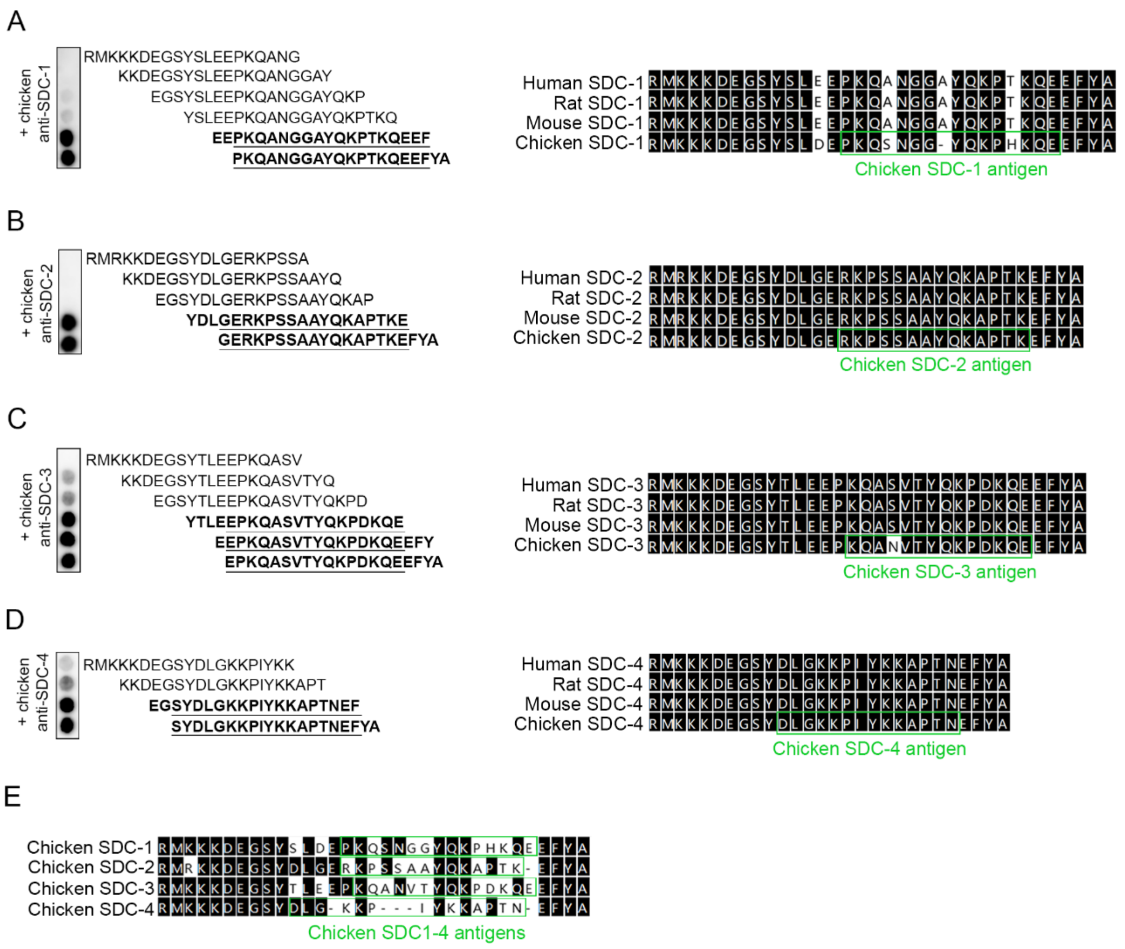

Figure S3. Epitope mapping of chicken syndecan-1 to -4 antibodies and syndecan-1 to -4 alignments across species. Chicken **(A)** SDC-1, **(B)** SDC-2, **(C)** SDC-3 and **(D)** SDC-4 antibodies were overlaid immobilized overlapping 20-mer synthetic peptides corresponding to the cytoplasmic part of SDC-1 to -4 (mouse/chicken). The core epitopes are underlined (from two independent peptide arrays). The chicken SDC-1-4 antigens are boxed in the alignments of human rat, mouse and chicken SDC-1-4$_{cyt}$ (left panels in A-D, respectively) (DNASTAR, Lasergene). SDC-1-4$_{cyt}$ were almost identical across the four species. **(E)** The specific chicken SDC-1-4 antigens are boxed in the alignment of chicken SDC-1-4$_{cyt}$ (DNASTAR, Lasergene). The alignment shows that each antigen is highly specific to each chicken SDC.

Figure S4

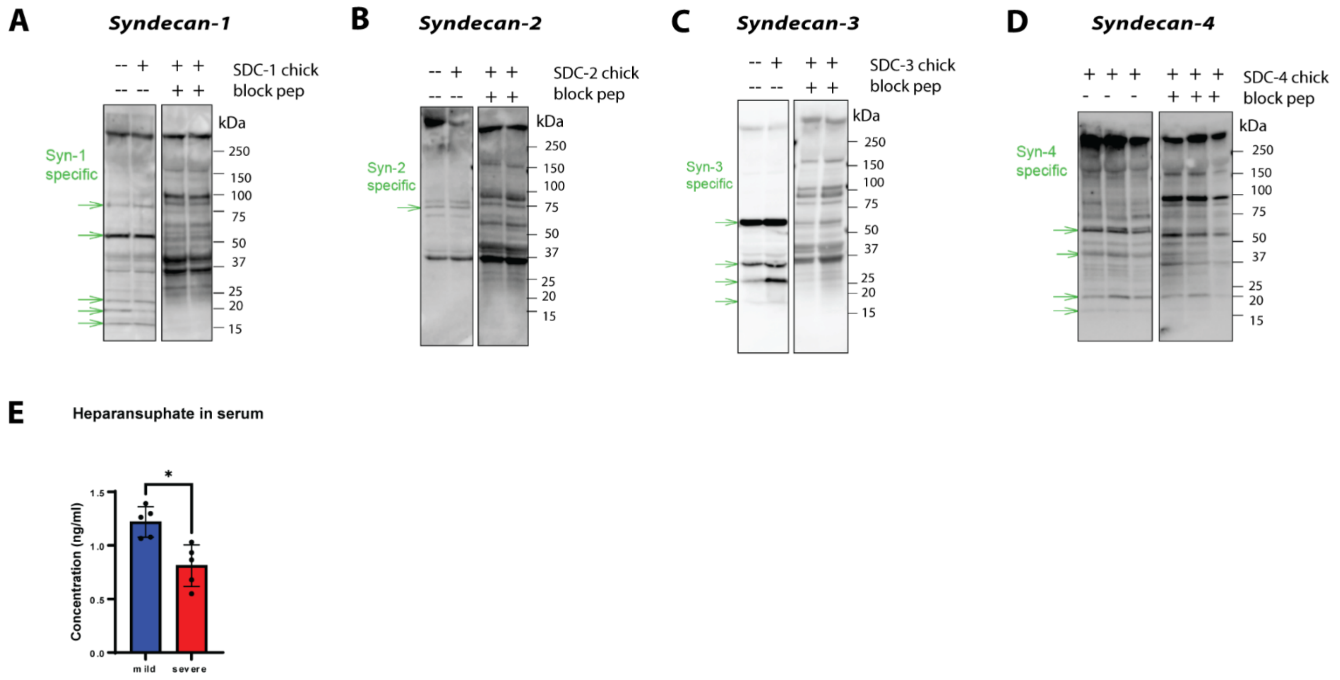

Figure S4. **Investigation of specific syndecans (A-D) bands by blocking peptides.** Representative samples were treated by syndecans antibodies and with or without a specific syndecan blocking peptide. Specific syndecan bands are highlighted by green arrows for each syndecan. **E)** Concentration of heparan sulfate in chicken serum. Chicken serum of mild samples was significantly increased compared to severe group. Results were obtained by competitive ELISA and calculated from standard curve by interpolation. Comparison between the groups was analyzed using one-way ANOVA using Brown-Forsythe and Welsh tests (* $p<0.05$).

Figure S5

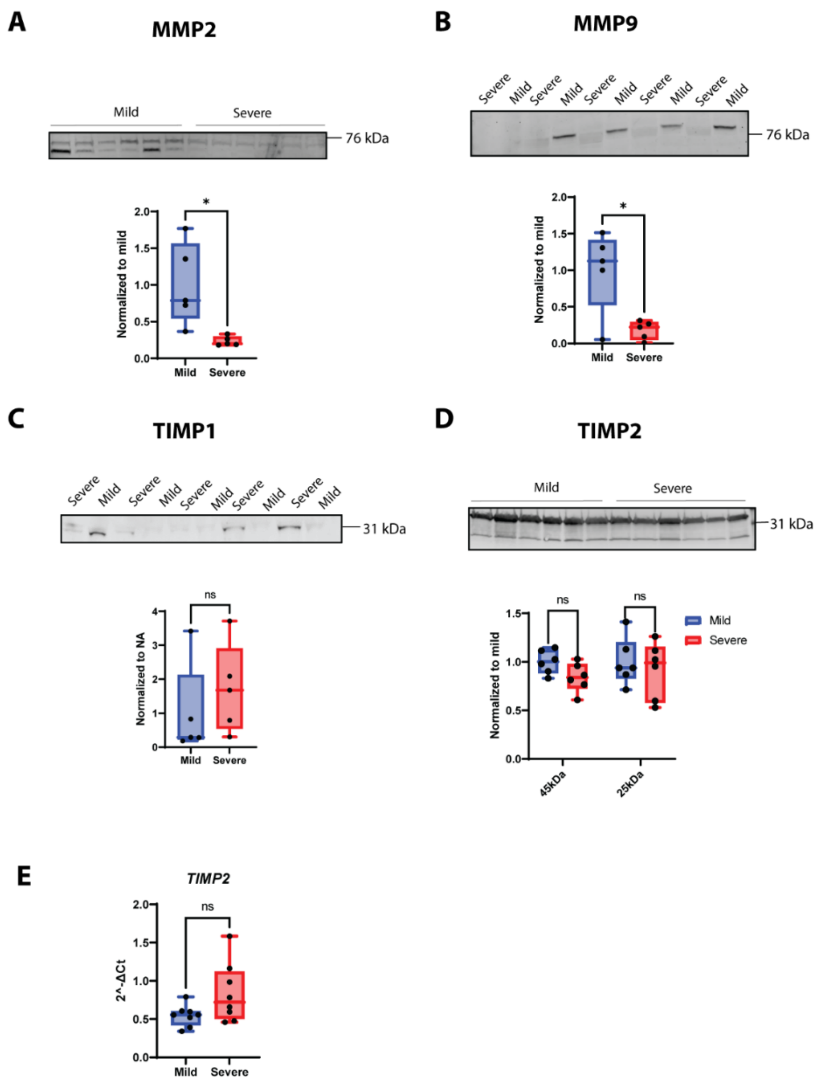

Figure S5. Protein expression of **(A)** MMP2 and **(B)** MMP9 are downregulated in severe WB. Protein expression of **(C)** TIMP1 and **(D)** TIMP2 remain unchanged between mild and severe WB groups, the same has been found for gene expression level of **(E)** TIMP2. Comparisons between the groups (mean ± SEM, n=8) were analyzed using t-test with Brown-Forsythe and Welsh correction (ns $p > 0.05$, * $p < 0.05$).

Table S1. Gene expression RNA Seq

	Gene	log2FoldChange*	Pvalue**	padj	Gene name
**Wnt signalling**	*APC*	0.806940913	2.11396E-09	4.43304E-08	APC WNT signaling pathway regulator
	APC2	0.03974368	0.917300987	0.94290881	APC2 WNT signaling pathway regulator
	AXIN1	-0.136235589	0.07174778	0.123464317	axin 1
	AXIN2	-0.147400421	0.340017167	0.449234951	axin 2
	CTNNA1	0.334916652	5.86625E-05	0.000235639	catenin alpha 1
	CTNNA2	-0.089844082	0.498079997	0.606605005	catenin alpha 2
	CTNNA3	0.024248769	0.846795001	0.89277416	catenin alpha 3
	CTNNAL1	0.853555699	7.94268E-07	5.5626E-06	catenin alpha like 1
	CTNNB1	0.076019582	0.10435857	0.170044881	catenin beta 1
	CTNNBIP1	-0.241423647	0.000439757	0.001406503	catenin beta interacting protein 1
	CTNNBL1	-0.185761925	0.011333525	0.02485168	catenin beta like 1
	CTNND1	0.31207272	0.000504818	0.0015872	catenin delta 1
	CTNND2	-0.696277483	0.009434729	0.02113513	catenin delta 2
	DACT1	0.959153693	1.65206E-09	3.62621E-08	dishevelled binding antagonist of beta catenin 1
	DACT2	-0.795324433	0.007028335	0.016297601	dishevelled binding antagonist of beta catenin 2
	DKK2	0.765268247	0.00085073	0.002522225	dickkopf WNT signaling pathway inhibitor 2
	DKK3	1.940390707	6.5595E-11	3.20528E-09	dickkopf WNT signaling pathway inhibitor 3
	DKK4	0.394001624	0.650900359	0.740372781	dickkopf WNT signaling pathway inhibitor 4
	DVL1	-0.388627764	1.14485E-06	7.63332E-06	dishevelled segment polarity protein 1
	DVL2	0.940404796	4.93705E-11	2.63343E-09	dishevelled segment polarity protein 2
	FZD1	1.652925612	4.71366E-13	1.09482E-10	frizzled class receptor 1
	FZD10	0.157943504	0.801696054	0.858946683	frizzled class receptor 10
	FZD3	0.139431879	0.427015794	0.537622671	frizzled class receptor 3
	FZD4	-0.185133267	0.028414785	0.0554191	frizzled class receptor 4
	FZD5	0.145885242	0.56455898	0.665494843	frizzled class receptor 5
	FZD6	-0.104713807	0.172023247	0.259769119	frizzled class receptor 6
	FZD7	-0.217457195	0.074792528	0.1279478	frizzled class receptor 7
	GSK3A	-0.225069343	0.002928596	0.007510385	glycogen synthase kinase 3 alpha
	GSKIP	-0.055107444	0.528242614	0.633635597	GSK3B interacting protein
	LRP1	1.093675466	2.48702E-12	3.12564E-10	LDL receptor related protein 1
	LRP11	0.123462735	0.44589081	0.556385615	LDL receptor related protein 11
	LRP12	-0.346088917	3.77827E-05	0.000159557	LDL receptor related protein 12
	LRP1B	-0.481857145	0.089863227	0.149804702	LDL receptor related protein 1B
	LRP2	1.037980072	0.000450531	0.001437955	LDL receptor related protein 2
	LRP2BP	-0.023267788	0.939326833	0.95775217	LRP2 binding protein

	LRP3	-0.07425766	0.504924196	0.612795428	LDL receptor related protein 3
	LRP4	1.488055237	4.19766E-08	4.81694E-07	LDL receptor related protein 4
	LRP5	-0.109714722	0.168710358	0.255502341	LDL receptor related protein 5
	LRP6	0.026778678	0.764433489	0.831145934	LDL receptor related protein 6
	LRP8	0.903733336	3.28906E-05	0.000141205	LDL receptor related protein 8
	MMP9	4.584979685	7.55963E-12	7.0412E-10	matrix metallopeptidase 9
	WNT4	0.819269418	0.010459846	0.023162891	Wnt family member 4
	WNT6	-0.098487286	0.816073648	0.869982301	Wnt family member 6
**Other signalling molecules**	*AKT1*	0.467678022	5.20193E-08	5.74309E-07	AKT serine/threonine kinase 1
	AKT3	0.17975004	0.012252858	0.026590577	AKT serine/threonine kinase 3
	AKTIP	-0.665154377	7.17692E-10	1.90302E-08	AKT interacting protein
	FGF2	1.006644387	9.80617E-08	9.60157E-07	fibroblast growth factor 2
	MTOR	0.06719778	0.225556823	0.324251187	mechanistic target of rapamycin
	NOTCH1	0.022552117	0.733411142	0.806326375	notch 1
	NOTCH2	0.462875344	1.43297E-05	6.7802E-05	notch 2
	RPS6	-0.231319404	0.0389918	0.073021017	ribosomal protein S6
	RPS6KA1	1.59976651	2.5063E-11	1.64832E-09	ribosomal protein S6 kinase A1
	RPS6KA2	0.959046552	3.39813E-06	1.95217E-05	ribosomal protein S6 kinase A2
	RPS6KA3	-0.269509984	0.004838785	0.011710216	ribosomal protein S6 kinase A3
	RPS6KA5	0.549046439	6.95251E-07	4.97161E-06	ribosomal protein S6 kinase A5
	RPS6KA6	-0.148292605	0.078065538	0.132849872	ribosomal protein S6 kinase A6
	RPS6KB1	-0.229618084	0.016135727	0.033833214	ribosomal protein S6 kinase B1
	RPS6KB2	-0.63346121	1.67622E-06	1.05706E-05	ribosomal protein S6 kinase B2
	RPS6KC1	-0.11972788	0.117145201	0.187499763	ribosomal protein S6 kinase C1
	RPS6KL1	0.707782793	0.000137103	0.000499744	ribosomal protein S6 kinase like 1
	RPS7	-0.227397551	0.023271135	0.0466465	ribosomal protein S7
	RPS8	-0.22950416	0.008095862	0.018448952	ribosomal protein S8
	SMAD2	-0.171306531	0.002529702	0.006598081	SMAD family member 2
	SMAD3	0.958111309	3.74593E-10	1.15505E-08	SMAD family member 3
	SMAD4	1.188601314	9.25497E-07	6.32946E-06	SMAD family member 4
	SMAD5	-0.284105283	0.002529258	0.006597861	SMAD family member 5
	SMAD6	-0.08521427	0.456947976	0.567327857	SMAD family member 6
	SMAD7	-0.080468114	0.461767465	0.572266055	SMAD family member 7
	SMAD9	0.093959146	0.468768542	0.578442096	SMAD family member 9
	TGFA	-0.353199784	0.110437852	0.178523843	transforming growth factor alpha
	TGFB1	0.848435847	4.739E-09	8.47523E-08	transforming growth factor beta 1
	TGFB2	0.210741166	0.02220952	0.044748269	transforming growth factor beta 2
	TGFB3	1.555924736	1.87259E-06	1.16278E-05	transforming growth factor beta 3

	TGFBR1	0.645144654	3.70343E-07	2.93159E-06	transforming growth factor beta receptor 1
	TGFBR2	0.824720446	1.04623E-05	5.15364E-05	transforming growth factor beta receptor 2
	TGFBR2L	1.255381268	3.90415E-09	7.26544E-08	transforming growth factor beta receptor 2 like
	TGFBR3	0.223260635	0.056820647	0.101105708	transforming growth factor beta receptor 3
**ECM molecules**	*ACTB*	0.729883613	7.60E-09	0.000	actin beta
	ACTN1	1.319324838	2.75E-11	0.000	actinin alpha 1
	ACTN2	-0.792624022	3.63E-06	0.000	actinin alpha 2
	BGN	0.088245578	0.564471982	0.66543504	biglycan
	DCN	1.548433256	3.76807E-12	4.06707E-10	decorin
	DMD	-0.095038558	0.479492143	0.58909952	dystrophin
	ELN	-0.116532898	0.671744008	0.758	elastin
	FGB	-0.267059937	0.529668358	0.635	fibrinogen beta chain
	FMOD	0.872105878	0.016538127	0.034546687	fibromodulin
	FN1	2.739512559	2.44E-12	0.000	fibronectin 1
	ITGA1	-0.557846101	5.05E-06	0.000	integrin subunit alpha 1
	ITGA11	1.007259926	5.37E-07	0.000	integrin subunit alpha 11
	ITGA2	0.653682448	0.005608371	0.013	integrin subunit alpha 2
	ITGA2B	0.381494672	0.044331874	0.081	integrin subunit alpha 2b
	ITGA3	0.570006517	2.44E-04	0.001	integrin subunit alpha 3
	ITGA4	0.452366097	0.002577215	0.007	integrin subunit alpha 4
	ITGA5	0.675799196	2.60E-04	0.001	integrin alpha 5 (fibronectin receptor alpha polypeptide)
	ITGA6	0.255967947	0.022732288	0.046	integrin subunit alpha 6
	ITGA7	-0.614700988	2.57E-06	0.000	integrin subunit alpha 7
	ITGA8	2.630019915	4.27E-14	0.000	integrin subunit alpha 8
	ITGA9	-0.552531564	5.35E-04	0.002	integrin subunit alpha 9
	ITGAD	-0.023454635	0.813685234	0.868	integrin alpha D
	ITGAV	0.696635542	1.13E-09	0.000	integrin subunit alpha V
	ITGB1	-0.192913012	0.002255407	0.006	integrin subunit beta 1
	ITGB1BP1	-0.116931245	0.387145669	0.498	integrin subunit beta 1 binding protein 1
	ITGB1BP3	-0.288238554	8.74E-05	0.000	integrin beta 1 binding protein 3
	ITGB2	1.898045376	1.70E-12	0.000	integrin subunit beta 2
	ITGB3	0.433359929	0.005713082	0.014	integrin subunit beta 3
	ITGB3BP	0.366406056	0.073966749	0.127	integrin subunit beta 3 binding protein
	ITGB4	0.925013131	1.56E-04	0.001	integrin subunit beta 4
	ITGB5	1.014118992	3.10E-12	0.000	integrin subunit beta 5
	ITGB6	0.045318887	0.846244795	0.892	integrin subunit beta 6
	ITGB8	1.909491768	6.52E-08	0.000	integrin subunit beta 8
	ITGBL1	1.379400121	2.08E-05	0.000	integrin subunit beta like 1
	LAMA1	-3.128211091	9.50E-08	0.000	laminin subunit alpha 1
	LAMA2	0.064832776	0.537837411	0.642	laminin subunit alpha 2
	LAMA3	0.435456059	0.020623995	0.042	laminin subunit alpha 3

	LAMA4	-0.347088585	0.001839105	0.005	laminin subunit alpha 4
	LAMA5	0.852603589	4.45E-08	0.000	laminin subunit alpha 5
	LAMB1	0.077738817	0.205674035	0.301	laminin subunit beta 1
	LAMB2	0.07255972	0.477885069	0.588	laminin beta 2 (laminin S)
	LAMB3	-0.070999474	0.772787752	0.838	laminin subunit beta 3
	LAMB4	-1.451171613	2.21E-06	0.000	laminin subunit beta 4
	LAMC1	-0.345215917	3.73E-05	0.000	laminin subunit gamma 1
	LAMC2	0.593018649	0.159518028	0.244	laminin subunit gamma 2
	LAMC3	-0.351404894	0.269786511	0.374	laminin subunit gamma 3
	LOX	1.409774936	1.24704E-09	2.87824E-08	lysyl oxidase
	LUM	1.594172017	4.11379E-13	9.83799E-11	lumican
	TNC	3.138803184	1.28E-13	0.000	tenascin C
	VTN	2.386353499	3.06E-09	0.000	vitronectin
**Syndecans and enzymes**	*ADAM11*	-2.228305201	6.53804E-10	1.76496E-08	ADAM metallopeptidase domain 11
	ADAM12	1.306826267	7.63371E-09	1.22762E-07	ADAM metallopeptidase domain 12
	ADAM33	0.956174238	6.6161E-09	1.10866E-07	ADAM metallopeptidase domain 33
	ADAM8	4.152289708	6.42558E-13	1.30666E-10	ADAM metallopeptidase domain 8
	HPSE	1.34542314	8.4312E-07	5.84893E-06	heparanase
	HPSE2	-1.14258821	1.16705E-07	1.1084E-06	heparanase 2 (inactive)
	MMP1	6.51346239	5.86185E-07	4.2972E-06	matrix metallopeptidase 1
	MMP10	4.472295844	5.19547E-07	3.87527E-06	matrix metallopeptidase 10
	MMP13	2.596211668	1.07507E-05	5.27728E-05	matrix metallopeptidase 13
	MMP15	-0.121178989	0.235336444	0.335810593	matrix metallopeptidase 15
	MMP16	0.758427429	1.95272E-06	1.20357E-05	matrix metallopeptidase 16
	MMP17	-0.823116895	4.63324E-06	2.53749E-05	matrix metallopeptidase 17
	MMP2	1.185036666	1.66266E-10	6.33603E-09	matrix metallopeptidase 2
	MMP24	0.651964677	2.14716E-06	1.30718E-05	matrix metallopeptidase 24
	MMP27	4.767164195	5.47242E-16	1.25517E-12	matrix metallopeptidase 1
	MMP28	0.239733061	0.075318533	0.128739614	matrix metallopeptidase 28
	MMP3	1.820919536	0.179744714	0.269631765	matrix metallopeptidase 3
	MMP7	4.372947762	3.56297E-09	6.74685E-08	matrix metallopeptidase 7
	SDC1	0.6298265	1.36546E-05	6.50943E-05	syndecan 1
	SDC2	-0.243677385	0.010895164	0.024034066	syndecan 2
	SDC3	-0.366298255	0.023124338	0.046392791	syndecan 3
	SDC4	0.921982623	1.27919E-05	6.13964E-05	syndecan 4
	TIMP2	0.726825784	9.16614E-08	9.05705E-07	TIMP metallopeptidase inhibitor 2
	TIMP3	-0.282539073	0.036695952	0.069280176	TIMP metallopeptidase inhibitor 3
	TIMP4	0.008910037	0.964696811	0.975865361	TIMP metallopeptidase inhibitor 4
**Collagens**	*COL10A1*	8.70E-02	0.940061026	0.958	collagen type X alpha 1
	COL11A1	3.86E+00	7.95E-13	0.000	collagen type XI alpha 1 chain
	COL12A1	3.80E+00	2.81E-14	0.000	collagen type XII alpha 1 chain
	COL13A1	5.29E-01	0.002038132	0.005	collagen type XIII alpha 1 chain

	COL14A1	1.13E+00	8.05E-08	0.000	collagen type XIV alpha 1 chain
	COL15A1	-9.93E-01	6.25E-08	0.000	collagen type XV alpha 1 chain
	COL16A1	1.71E+00	5.72E-11	0.000	collagen type XVI alpha 1 chain
	COL17A1	0.69	0.250589483	0.352964338	collagen type XVII alpha 1 chain
	COL18A1	-5.06E-01	7.52E-08	0.000	collagen type XVIII alpha 1
	COL19A1	6.24E-01	0.003471086	0.009	collagen type XIX alpha 1 chain
	COL1A1	2.34E+00	5.57E-08	0.000	collagen type I alpha 1 chain
	COL1A2	2.23E+00	1.40E-08	0.000	collagen type I alpha 2 chain
	COL20A1	-5.66E-01	7.58E-04	0.002	collagen type XX alpha 1 chain
	COL21A1	3.39E-01	0.039028962	0.073	collagen type XXI alpha 1 chain
	COL22A1	-2.55E-01	0.296392505	0.403	collagen type XXII alpha 1 chain
	COL23A1	1.11E+00	2.81E-07	0.000	collagen type XXIII alpha 1 chain
	COL24A1	4.16E-01	0.189795746	0.282	collagen type XXIV alpha 1 chain
	COL25A1	1.87E+00	5.93E-08	0.000	collagen type XXV alpha 1 chain
	COL26A1	-3.18E-01	0.094902477	0.157	collagen type XXVI alpha 1 chain
	COL27A1	8.59E-01	1.47E-06	0.000	collagen type XXVII alpha 1 chain
	COL28A1	-7.99E-01	8.46E-08	0.000	collagen type XXVIII alpha 1 chain
	COL2A1	-7.50E-02	0.946674055	0.963	collagen type II alpha 1 chain
	COL3A1	1.43E+00	1.06E-08	0.000	collagen type III alpha 1 chain
	COL4A1	-7.21E-01	7.43E-11	0.000	collagen type IV alpha 1 chain
	COL4A2	-7.42E-01	3.87E-09	0.000	collagen type IV alpha 2 chain
	COL4A3	1.47E+00	2.02E-07	0.000	collagen type IV alpha 3 chain
	COL4A4	1.59E+00	2.14E-06	0.000	collagen type IV alpha 4 chain
	COL4A5	8.59E-01	3.81E-06	0.000	collagen type IV alpha 5 chain
	COL4A6	8.45E-01	3.57E-07	0.000	collagen type IV alpha 6 chain
	COL5A1	1.46E+00	3.01E-10	0.000	collagen type V alpha 1 chain
	COL5A2	1.15E+00	4.65E-09	0.000	collagen type V alpha 2 chain
	COL6A1	9.66E-01	9.55E-09	0.000	collagen type VI alpha 1 chain
	COL6A2	9.76E-01	1.46E-09	0.000	collagen type VI alpha 2 chain
	COL6A3	1.17E+00	8.26E-10	0.000	collagen type VI alpha 3 chain
	COL6A6	2.05E+00	7.80E-08	0.000	collagen type VI alpha 6 chain
	COL7A1	2.09E+00	5.52E-11	0.000	collagen type VII alpha 1
	COL8A1	9.67E-01	2.72E-07	0.000	collagen type VIII alpha 1 chain
	COL8A2	2.13E+00	4.94E-12	0.000	collagen type VIII alpha 2 chain
	COL9A1	-1.084	0.120	0.192	collagen type IX alpha 1 chain
	COL9A2	1.75E+00	8.76E-09	0.000	collagen type IX alpha 2 chain
	COL9A3	8.15E-01	0.02175176	0.044	collagen type IX alpha 3 chain

**Immuno markers**	*IL10*	-0.20798371	0.948782167	NA	interleukin 10
	IL1B	1.258184962	0.017431258	0.036214465	interleukin 1 beta
	IL6	3.550116066	0.001322053	0.003723461	interleukin 6
	TLR4	1.579610581	3.97236E-09	7.36251E-08	toll like receptor 4
	VCAM1	0.282925468	0.11288163	0.181753688	vascular cell adhesion molecule 1

* log2 fold change represents the difference between the levels of expression for each gene between severe WB and mild WB groups.
** Significant pvalue p<0.05 is highlighted by orange color.

Raw data western blotting, SDS-PAGE

All presented gels show frames for cropped areas presented in the manuscript. The difference between the group of samples is made by blue line dividing mild WB (left side) samples and the severe WB samples (right side). The standards of molecular weights on western blots are presented on the both sides in kDa (250,150,100, 75, 50, 37, 25, 20, 15, (10 – not present in cropped blots)).

Figure 4

Total protein Ponceau S:

E) Syndecan-1

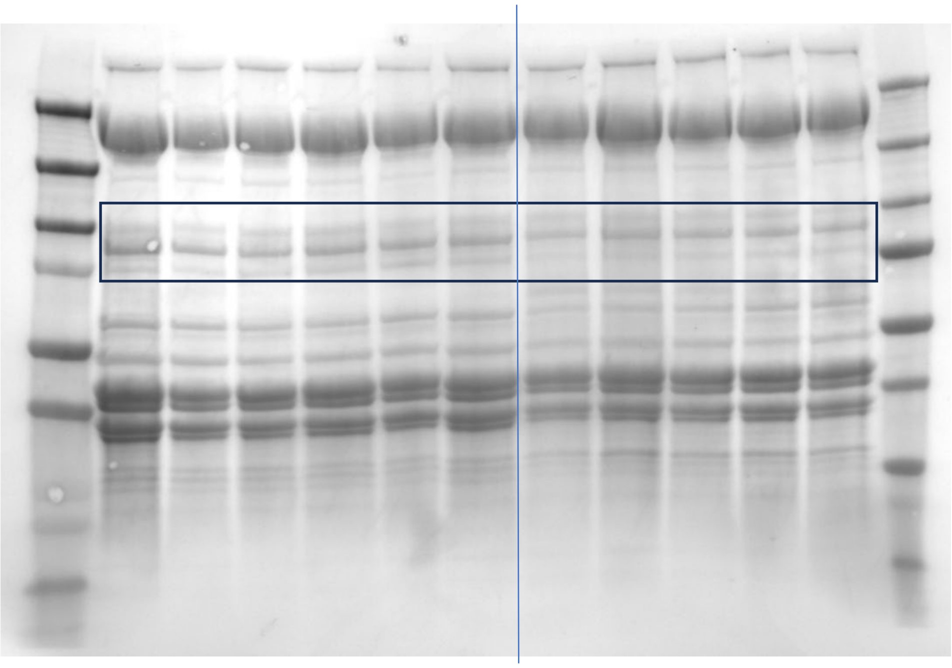

F) Syndecan-2

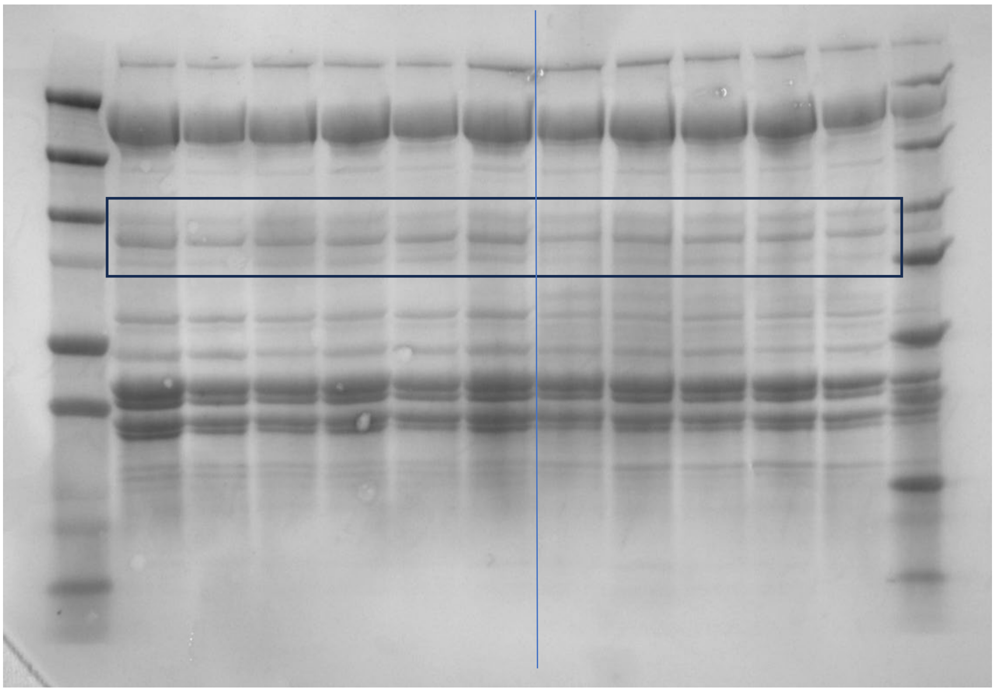

G) Syndecan-3

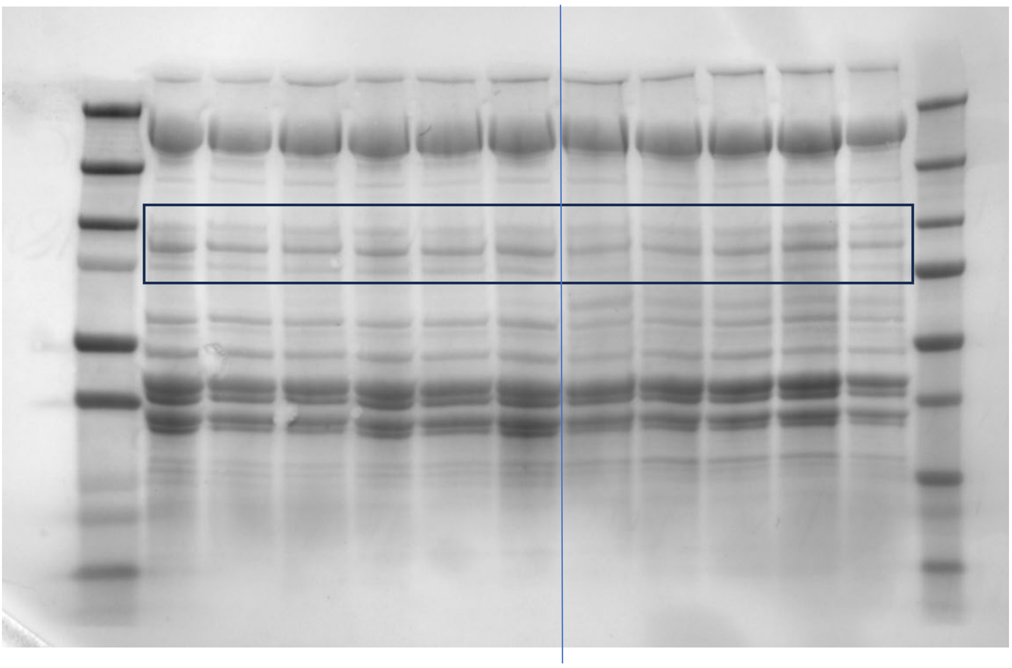

H) Syndecan-4

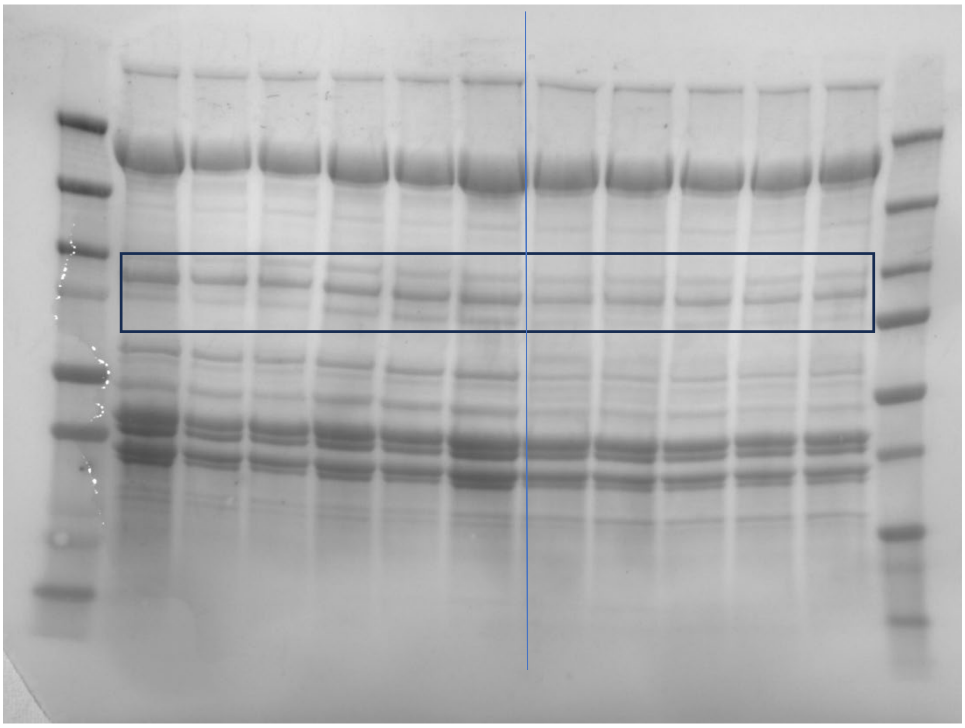

Figure 5

C) Zymography

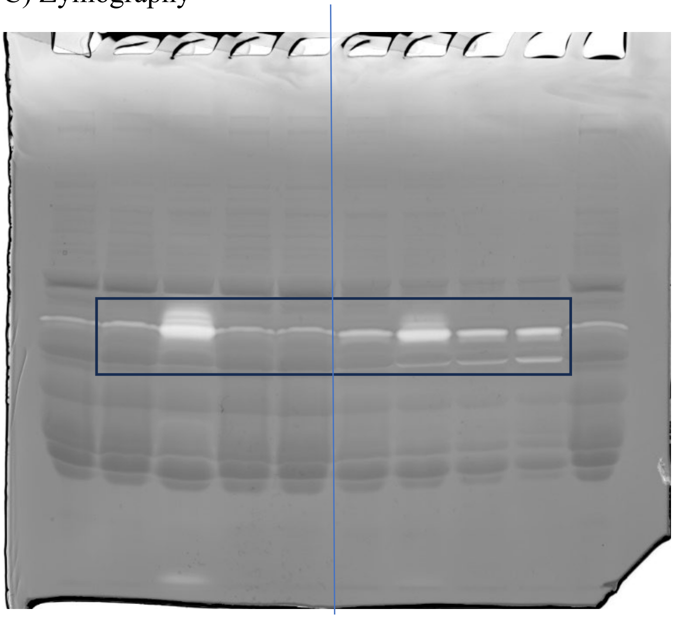

The left and the right line of SDS-PAGE show reference mild sample used on all zymography gels to keep constant reference. In the quantification was then included only once, and this gel exclude these controls in cropped version.

Figure 6

A) ERK1/2 total, pThr202/Tyr204-ERK1/2

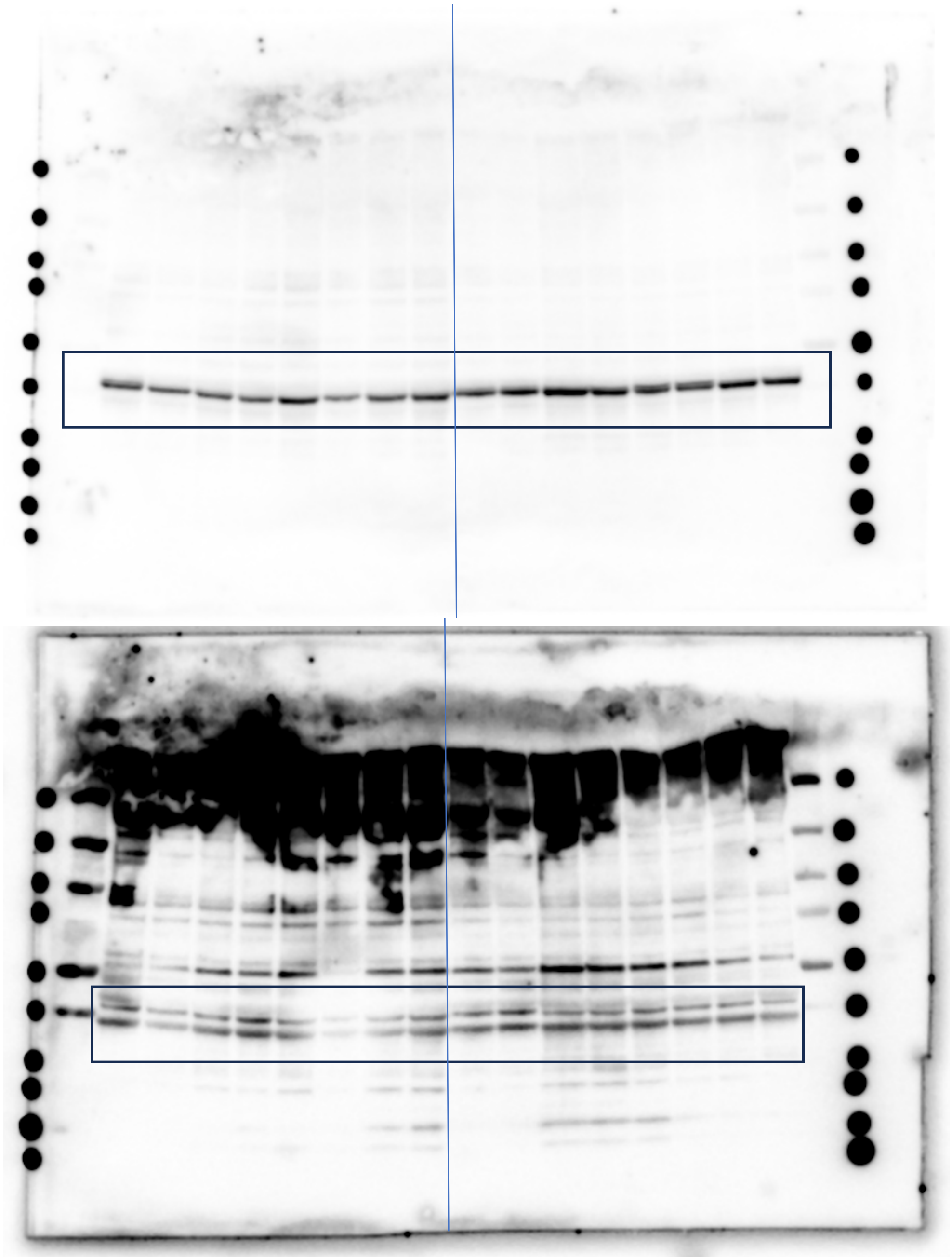

Total protein Ponceau S:

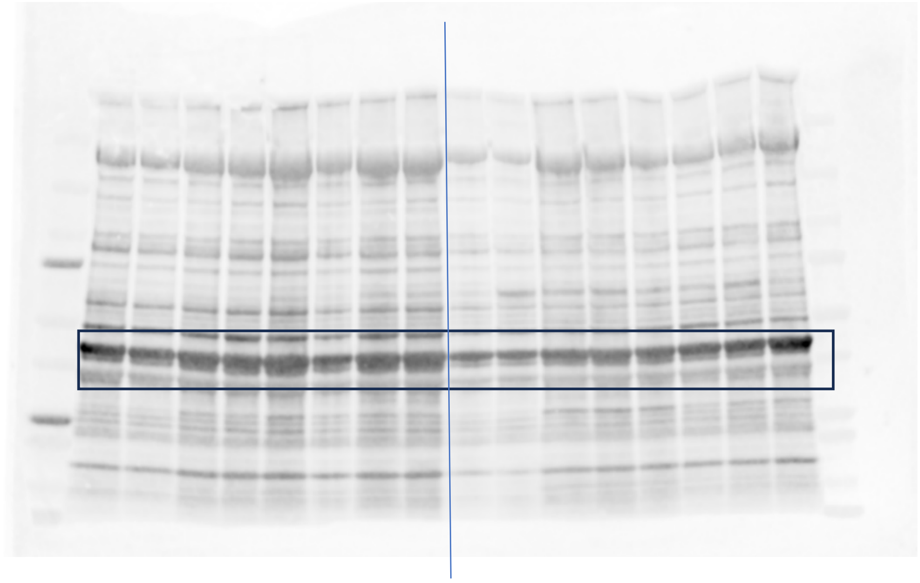

B) AKT total, pSer473-AKT

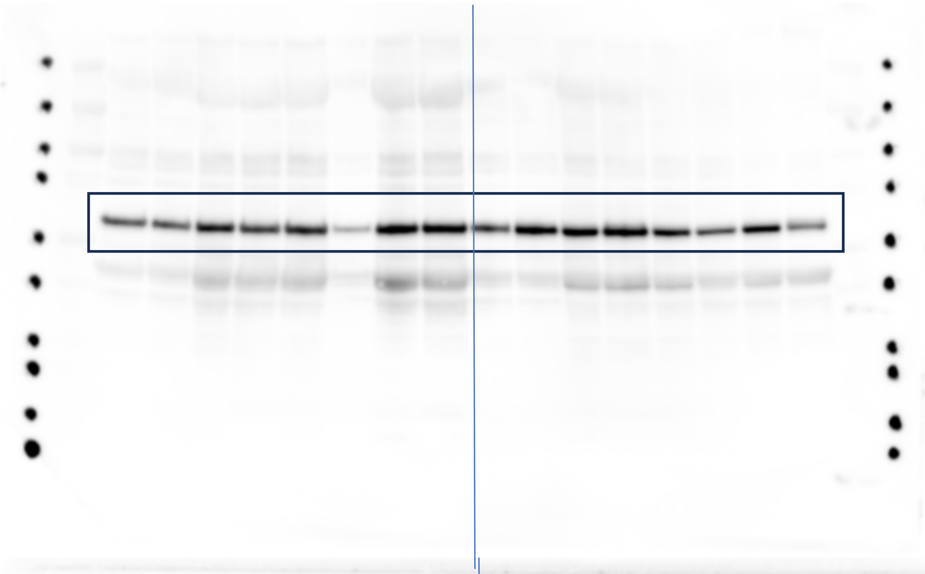

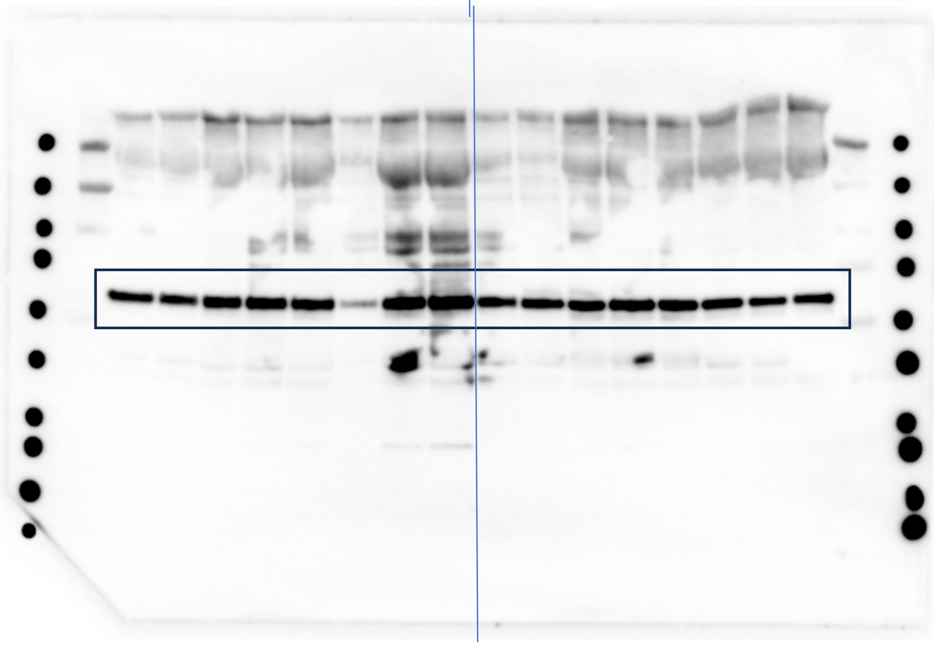

Total protein Ponceau S:

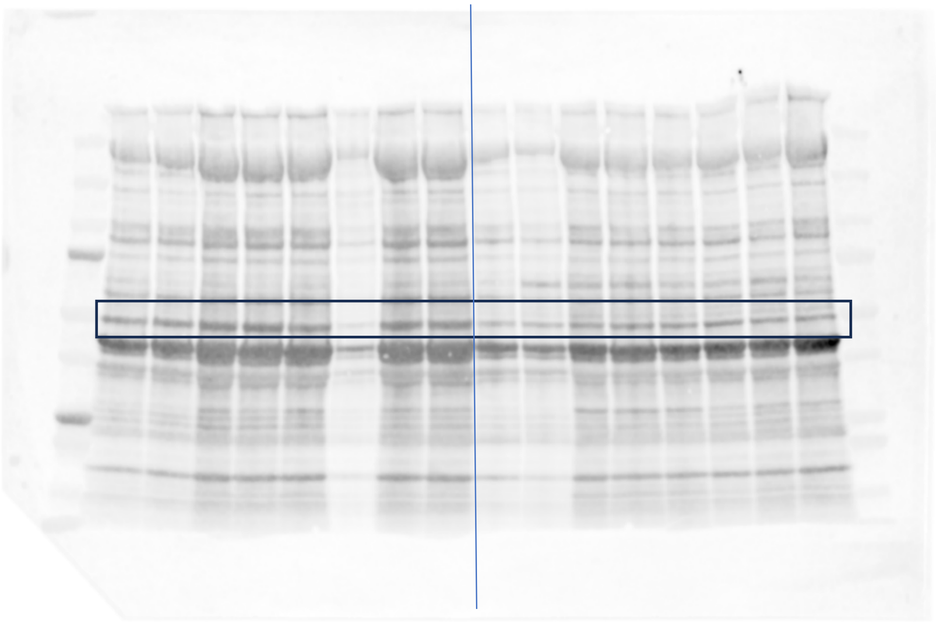

C) rpS6 total, pSer240/244-rpS6

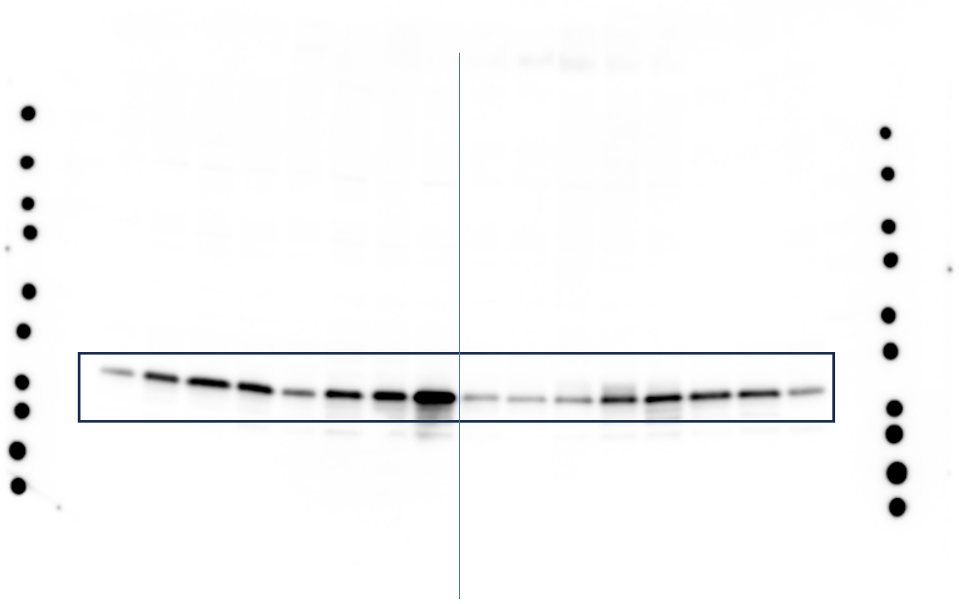

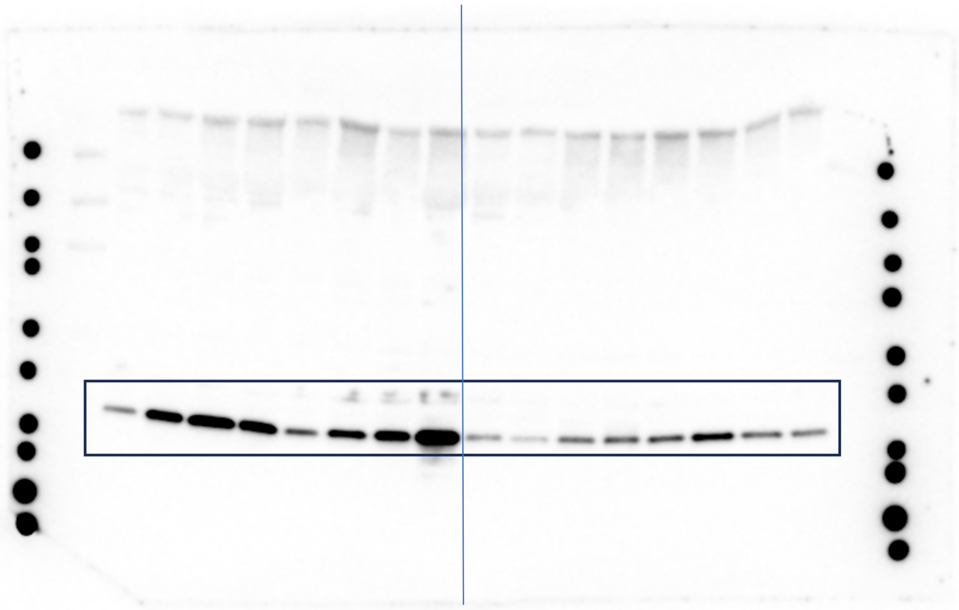

Total protein Ponceau S:

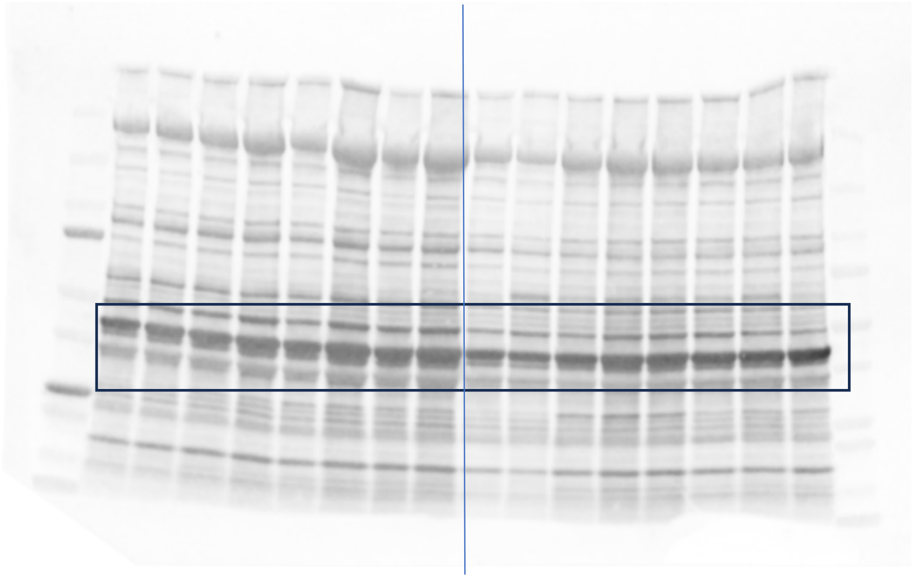

Figure 7

A) Beta-catenin

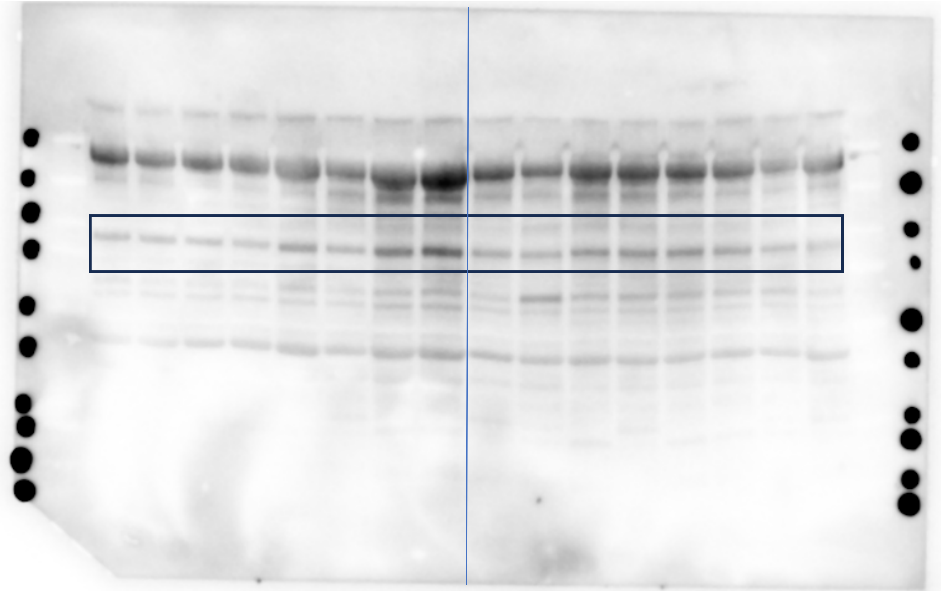

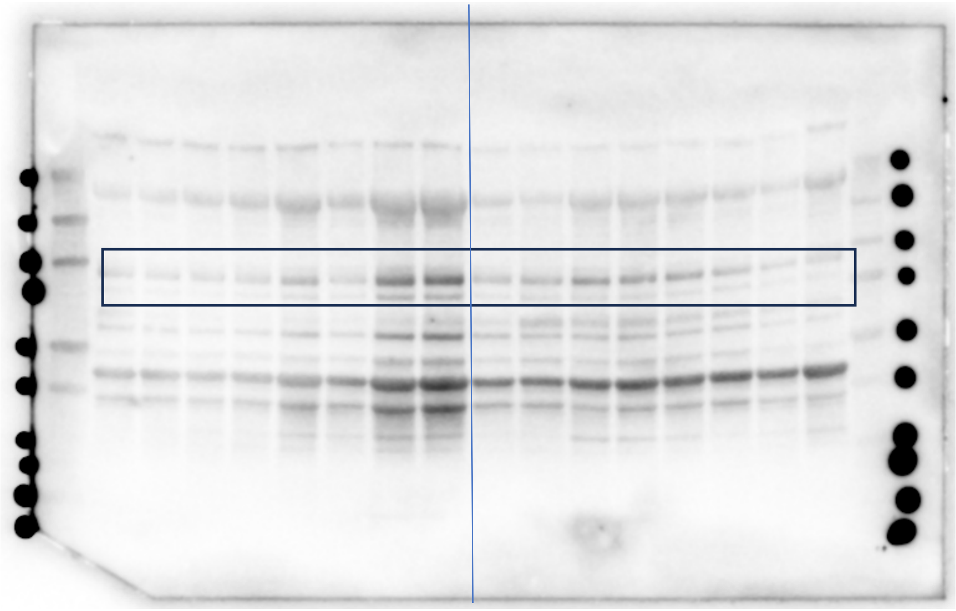

Total protein Ponceau S:

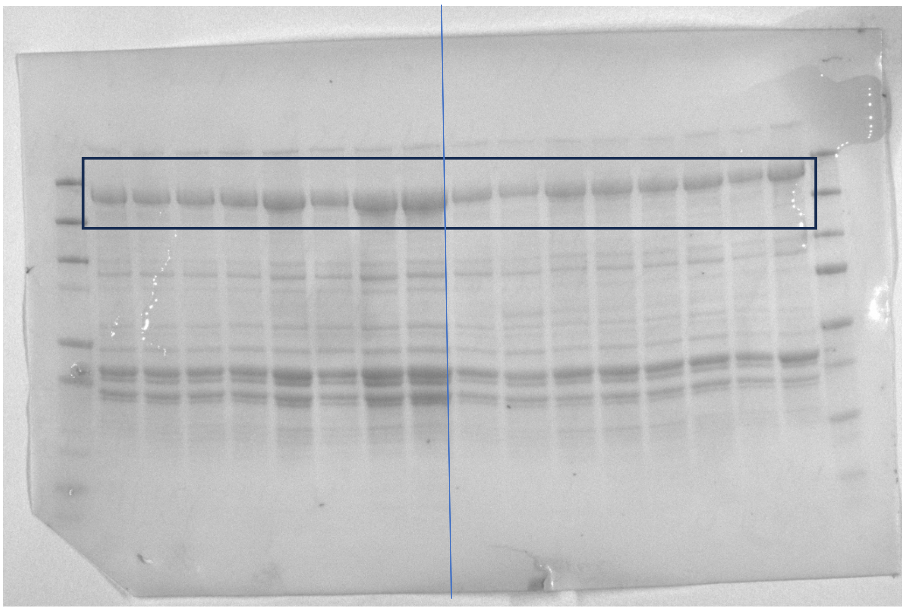

B) Wnt4

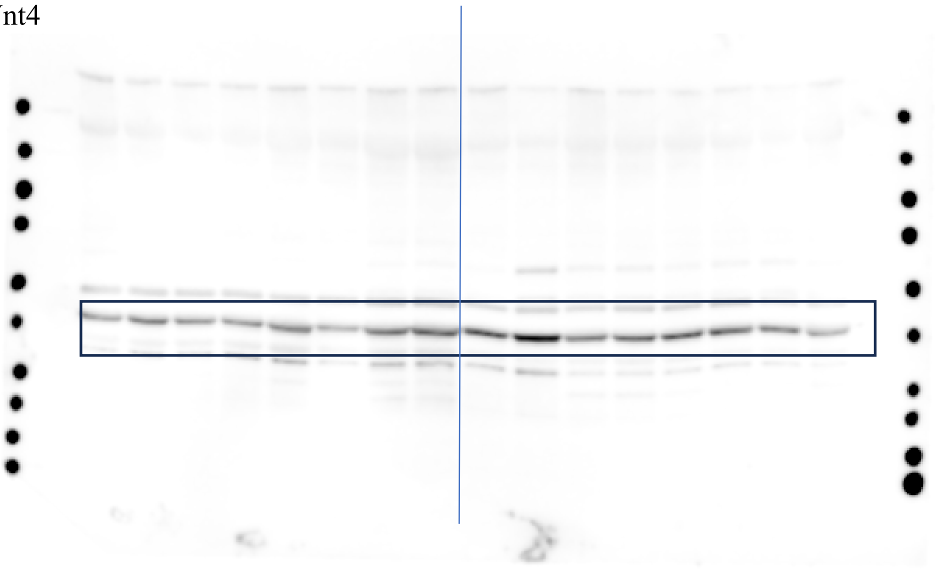

Total protein Ponceau S:

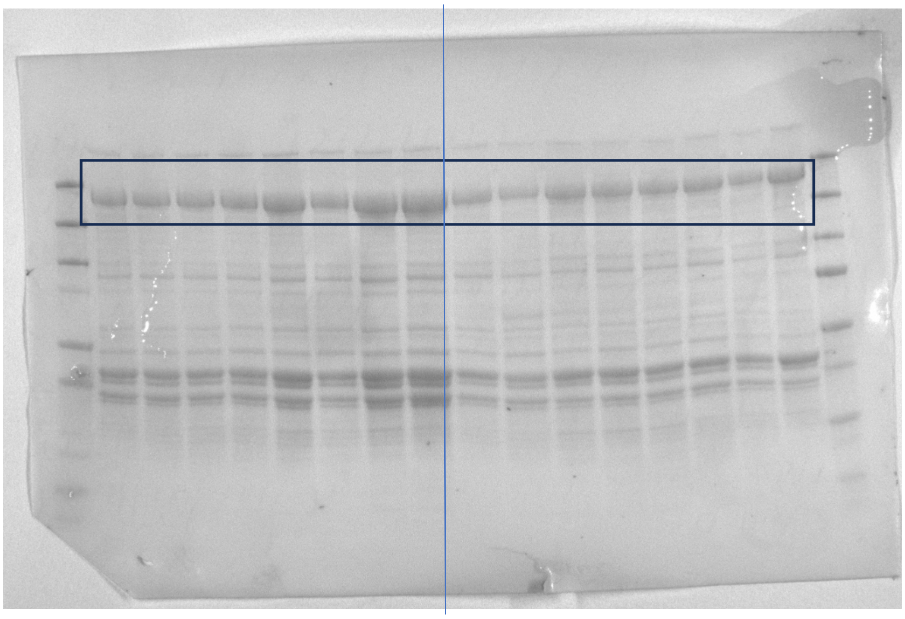

C) Wnt7a

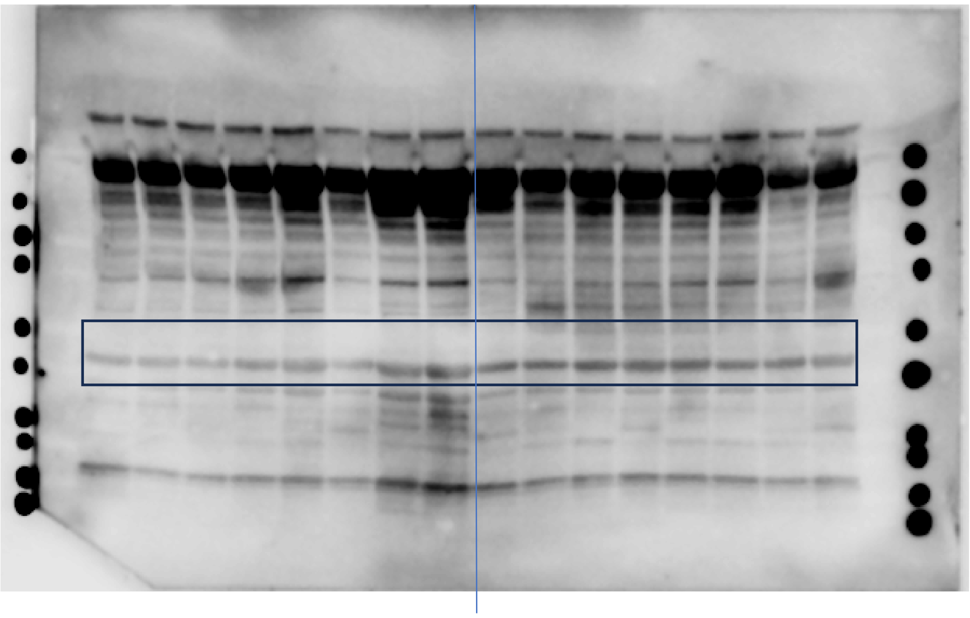

Total protein Ponceau S:

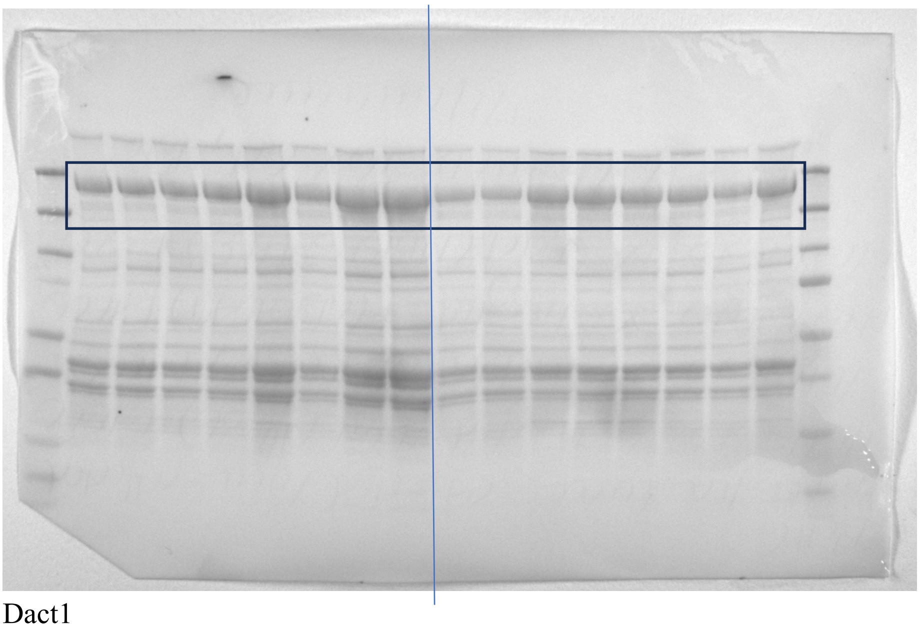

D) Dact1

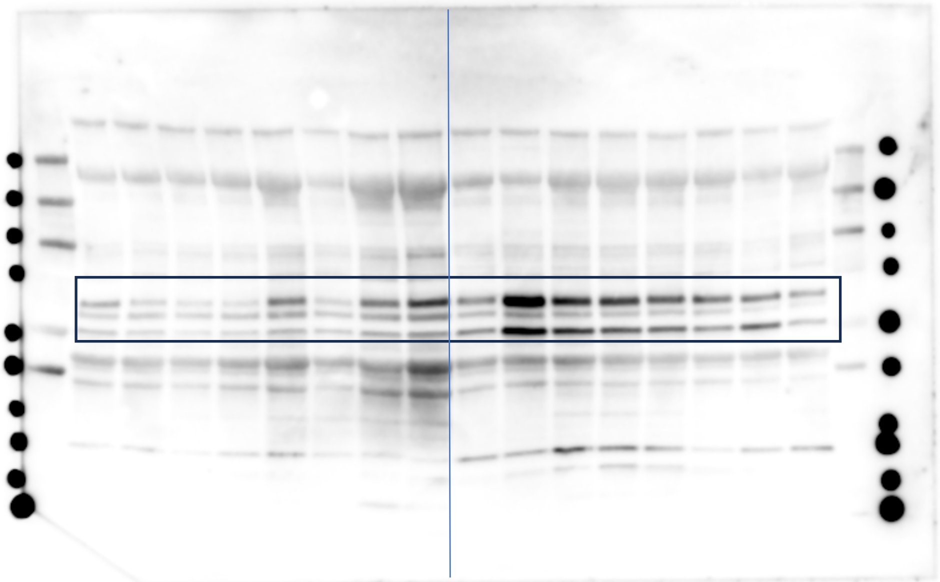

Total protein Ponceau S:

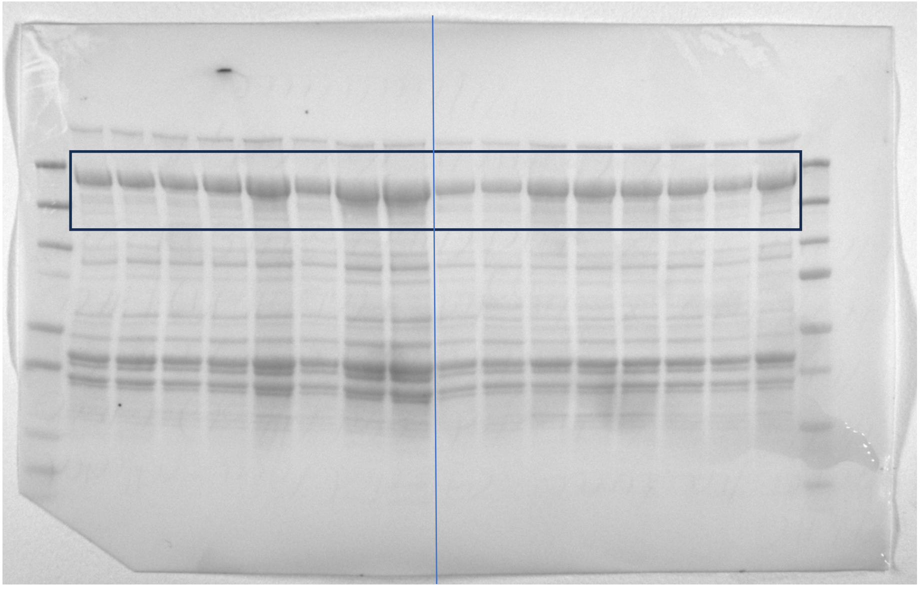

Supplement: Supplementary file 1 [file DataSheet1.pdf]
